# Sedimentological and petrophysical characterization of the Bokabil Formation in the Surma Basin for CO_2_ storage capacity estimation

**DOI:** 10.1038/s41598-024-66373-7

**Published:** 2024-07-16

**Authors:** Shakhawat Hossain, Naymur Rahman, Himadri Shekhar

**Affiliations:** 1 Department of Geology, University of Dhaka, Dhaka, 1000 Bangladesh; 2 Department of Earth Science and Engineering, Imperial College London, London, UK

**Keywords:** Bokabil Formation, CO_2_ storage, Petrophysics, Petrography, Cap rock, Surma Basin, Carbon capture and storage, Environmental impact

## Abstract

Large-scale geological sequestration of CO_2_ is one of the most effective strategies to limit global warming to below 2 °C, as recommended by the Intergovernmental Panel on Climate Change (IPCC). Therefore, identifying and characterizing high-quality storage units is crucial. The Surma Basin, with its four-way dip closed structures, high-quality reservoirs, and thick regional cap rocks, is an ideal location for CO_2_ storage. This study focuses on the Bokabil Formation, the most prominent reservoir unit in the Surma Basin. Detailed petrographic, petrophysical, XRD, and SEM analyses, along with mapping, have been conducted to evaluate the properties of the reservoir and cap rock within this formation. The Upper Bokabil Sandstone in the Surma Basin ranges from 270 to 350 m in thickness and consists of fine- to medium-grained subarkosic sandstones composed of 70–85% quartz and 5–12% feldspar, with good pore connectivity. Petrophysical analysis of data from four gas fields indicates that this unit has a total porosity of 21–27.4% and a low shale volume of 15–27%. Cross plots and outcrop observations suggest that most of the shales are laminated within the reservoir. The regional cap rock, known as the Upper Marine Shale (UMS), ranges in thickness from 40 to 190 m and contains 10–40 nm nano-type pores. A higher proportion of ductile materials with a significant percentage of quartz in the UMS indicates higher capillary entry pressures, enhancing its capacity to hold CO_2_. Using the CSLF method with a 6% cut-off of the available pore volume, it is estimated that 103 Mt, 110 Mt, 205 Mt, and 164 Mt of CO_2_ can be effectively stored in the Sylhet, Kailashtila, Habiganj, and Fenchuganj structures, respectively. Due to the shallow depth of the storage unit and the thick cap rock, the southern Surma Basin is the optimal location for CO_2_ injection.

## Introduction

In the last decades, the growing concern over climate change and its impacts have centered on the escalating levels of carbon dioxide (CO_2_) in the atmosphere^1–5^. From 1870 to 2015, the cumulative release of anthropogenic CO_2_ into the atmosphere was approximately 2035 ± 205 Gigatonnes^6^. The concentration of CO_2_ in the pre-industrial revolution was about 280 ppmv (part per million by volume) and at present it increased to 395 ppmv^7^. These increased concentrations of CO_2_ are the driving force behind climate change^8–12^. To mitigate catastrophic climate changes and limit global temperature rise to below 2 °C, an 80% reduction in greenhouse gas emissions by 2050 is imperative^13–15^. Achieving this goal necessitates significant reductions in CO_2_ emissions and the capture and sequestration of CO_2_ from point sources. Therefore, a collective effort is needed from all participating countries and do their part for CO_2_ emission reductions as a commitment to the Paris agreement 2015^16^.

Bangladesh is one of the most rapidly developing countries in the world and at the same time one of the most vulnerable countries to the climate change due to the geographic location and low elevation^17–19^. Because of the increasing demand of energy to power the growth in the economy several new coal-based powerplants and industries are being developed and they will inevitably contribute to the emissions of CO_2_ in the atmosphere. In 1946, Bangladesh annually released approximately 0.12 Mt of CO_2_ into the atmosphere^20^. After 40 years, in 1986, this emission surged to 11.46 Mt, nearly 100 times of the 1946 level^20^. By 2000, the annual emission reached 26.52 Mt, and in 2021, it soared to 93.18 Mt^20^. Specifically, in 2019, 39.48 Mt of CO_2_ were released from the electricity and heat production sector, 24.93 Mt from the manufacturing and construction sector, 11.66 Mt from transportation, and 9.8 Mt from buildings^21^. Recently, Bangladesh has been completing three large coal-based power plant projects, namely the 1320 MW Rampal Power Station, 1200 MW Matarbari Power Station, and 1320 MW Payra Thermal Power Plant. Approximately 1023.758 kg of CO_2_ is produced to generate 1 MW of electricity from a coal-based power plant^22^. Therefore, a significant amount of CO_2_ will be released annually into the atmosphere from these three coal-based power plants and consequently contribute to global warming. To achieve the net zero emission target, it is imperative to capture these CO_2_ and inject in the subsurface.

Being one of the thickest sedimentary basins in the world, the Bengal Basin boast of having some of the most laterally extensive reservoirs. The Miocene aged fluvio-deltaic Bokabil Formation is one of them and considered as the most prolific reservoirs in the Bengal Basin. In the Bangladesh part of the Bengal Basin, 28 gas fields have been discovered thus far, estimated to possess a total recoverable gas reserve of 838.405 BCM (29.608 TCF)^23,24^. Among these fields, 11 are situated in the Surma Basin, containing an estimated total recoverable gas reserve of about 304.916 BCM (10.768 TCF)^23,25^. As of 2021, approximately 541.135 BCM (19.11 TCF) of the gas reserves have been extracted in the Bengal Basin^24^. Presently, Bangladesh consumes around 28.317 BCM (1 TCF) of natural gas annually, and the production rate from existing gas fields is approximately 25.485 BCM (900 BCF) per year, suggesting that commercial natural gas production in existing fields will continue for the next 10 years, after which the reservoirs will deplete and be abandoned at some point^24^. These gas fields, along with the existing infrastructure, present significant opportunities to contribute to the journey toward achieving net-zero emissions and create potential for CO_2_ trading in Bangladesh. Globally, many efforts are ongoing to develop fluvio-deltaic reservoirs as CO_2_ storage unit such as Bunter Sandstone Formation in the UK^26–29^, the Buntsandstein sandstone unit in the Netherlands^30,31^, Dupuy and Katnook Formation in Australia^32–34^. The findings from this study will be applicable in other places with similar geological setting.

The objective of this study is to characterize the Bokabil Formation within the Surma basin regarding its CO_2_ storage capacity in four gas fields. Additionally, the regional seal rock known as the Upper Marine Shale (UMS) within the Bengal Basin is part of this formation and remains uncharacterized to date. Here, we integrated detailed sedimentological and subsurface geophysical data, and used well-established reservoir characterization workflows to characterize the Bokabil reservoir and identify how much CO_2_ can be stored in this formation in the studied four gas fields. Based on the available pore volume and cap rock integrity the most optimal location for storing CO_2_ has been identified.

## Regional geology and stratigraphy

Bangladesh, located in the eastern south Asia occupies a large portion of the Bengal Basin, and the basin is bounded in the north by Shillong plateau, in the east by Indo-Burman ranges, in the west by Indian shield and in the south open to Bay of Bengal. The Bengal basin is a well-known sedimentary basin for almost 22 km Cretaceous to Holocene thickest sedimentary succession with prolific hydrocarbon bearing and potential hydrocarbon generation, accumulation in the world^35–37^. The Bengal Basin initiated its development during early Cretaceous time by the breakup of Gondwana supercontinent and the evolved Indian plate started to move northward with an Atlantic type passive margin in the eastern margin of the plate. After the rift tectonic phase, the Indian plate collided with the Eurasian plate in the north and the Burma plate in the east and started to subduct beneath those plates since the late Eocene time^38^. The continuous interaction through Eocene to Holocene time among the Indian plate, Eurasian plate, and Burma plate resulted the current configuration of the Bengal Basin^38–40^. The Bengal basin as well as Bangladesh containing 6 major tectonic features, such as: Chittagong-Tripura Fold belt, Bengal Foredeep, Calcutta-Mymenshing Hing zone, Bogra Shelf, Rangpur Platform and Sub-Himalayan Foredeep^41^. The studied wells are located and scatted in the Surma Basin and the Surma Basin covering a large portion of Bengal Foredeep and a portion of northern Chittagong-Tripura Fold belt.

**Figure 1:**
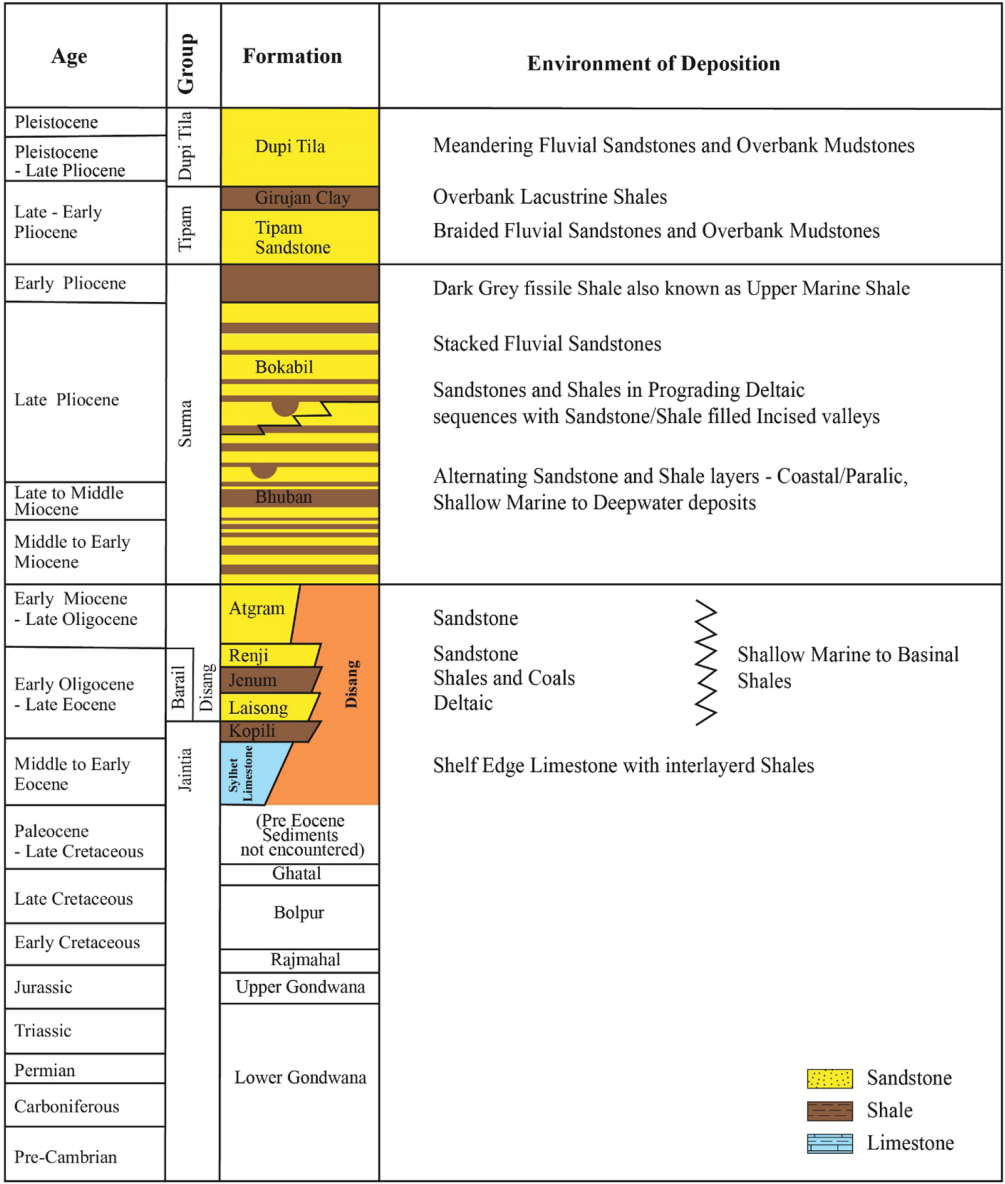
Stratigraphic succession of the Surma Basin showing all the stratigraphic units^23^. The stratigraphic succession was created using Adobe Illustrator CC 2021 (https://www.adobe.com/uk/products/illustrator.html).

The Surma Basin covers the north eastern part of Bangladesh with an area about 13,500 km^2^ and located at the Junction of Indian plate, Eurasian plate, and Burma plate^41^. The stratigraphy of Surma Basin is almost analogous to Assam stratigraphy of India^42,43^. The Surma Basin stratigraphy (Fig. 1) comprised with a few major formations, such as: Tura sandstone, Sylhet limestone, Kopili shale, Jenum, Renji, Bhuban, Bokabil, Tipam and Dupi Tila, Dihing and Alluvium Formation^43–46^. The Sylhet Limestone, Kopili shale and Jenum formations are the potential major source rocks of the Surma Basin^47–49^. The studied Upper Bokabil Sandstone unit of Bokabil Formation in Surma Basin is the most significant hydrocarbon bearing unit in the Surma Basin as well as Bengal Basin^23,50–52^, which comprises medium to fine grained, moderately sorted, trough cross bedded sandstone with a few isolated mud drapes and defined the depositional environment as a braided fluvial system^53^. The regionally extensive Upper Marine Shale unit of Bokabil Formation is the main top seal unit which comprises predominantly dark gray to black shale with a little amount silt and indicating marine transgression over Sylhet as well as other parts of the Bengal Basin^54,55^.

## Data and methodology

### Sedimentological analysis

Detailed sedimentological analyses were conducted to identify the contacts of the various stratigraphic units, nature of the reservoir and seal rocks. Petrographic analysis of the collected samples was performed for the identification of texture, mineralogy, and porosity. In total 14 samples were prepared for sieve and petrographic analysis. The samples were collected from the exposed outcrops of the Bokabil Formation along the Shari River cut section^53^.

### Petrophysical analysis

**Figure 2:**
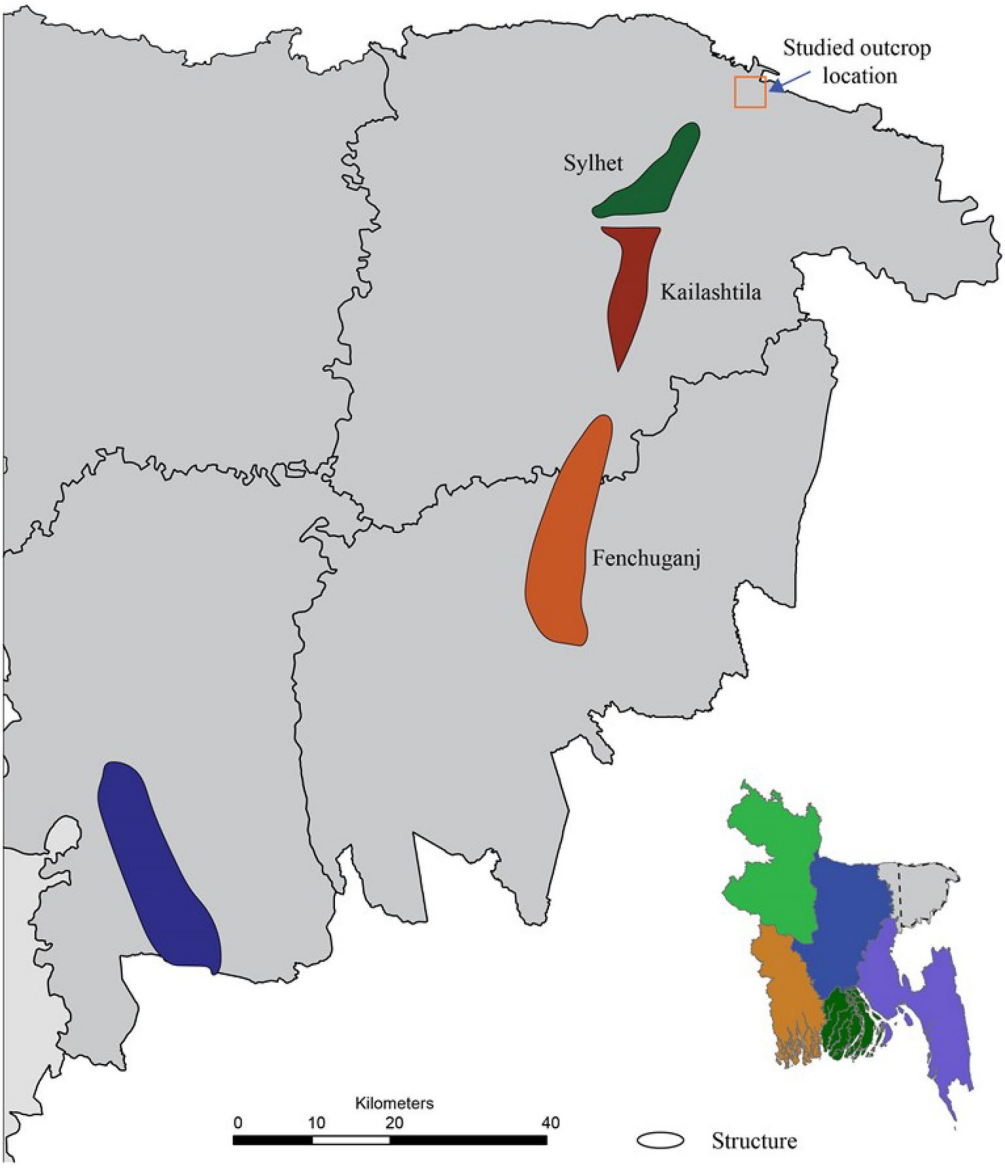
Map of Bangladesh in the inset showing the extent of study area. Location of the gas fields within Surma basin and the outcrop location^52^. The map was created using Adobe Illustrator CC 2021 (https://www.adobe.com/uk/products/illustrator.html).

Wells from Sylhet, Kailashtila, Habiganj and Fenchuganj gas fields (Fig. 2) have been selected to study spatial variation of petrophysical properties of Bokabil Formation in the Surma Basin. The petrophysical analysis of the well logs from these gas fields had been carried out by many authors^52,56–59^, However those studies were general, brief, and not focused on the Bokabil Formation. Therefore, the qualitative and quantitative analysis have been carried out by the available well logs data in a consistent manner. The used geophysical well logs are caliper (CAL), gamma ray (GR), resistivity shallow (MSFL) and deep (ILD), density (DEN), neutron (NPHI), compressional sonic (DT) and spontaneous potential (SP) logs (Table 1).

**Table 1:** The available wireline log responses of four wells scattered in the Surma Basin.

Well name	CAL (inches)	GR (API)	Resistivity (Ωm)		DEN (g/cm^3^)	NPHI (m^3^/m^3^)	DT (us/ft)	SP (mV)
			MSFL	ILD				
Sylhet	Y	Y	N	Y	Y	Y	Y	N
Kailashtila	Y	Y	Y	Y	Y	Y	Y	Y
Habiganj	Y	Y	Y	Y	Y	Y	Y	N
Fenchuganj	Y	Y	N	Y	Y	Y	Y	N

Here, Y and N denoted for availability and non-availability of the respective log data.

The Python libraries, namely Numpy, Pandas, and Matplotlib have been used for the various types of well log analysis and visualization including petrophysical and petrographic analysis (Supplementary information). The essential geophysical well logs are de-spiked as well as removed outliers by using OneClassSVM submodule of SVM (Support Vector Machine) module of unsupervised python machine learning techniques. The OpenCV python machine learning library has been used for petrographic analysis by converting color image to gray level image, transforming image from 2 to 1D and then segmenting the image based on 1D histogram.

#### Volume of Shale (V_sh_) determination

Volume of Shale (V_sh_) calculation is essential because it helps to differentiate between reservoir and seal rock, to calculate porosity, permeability, fluid content and overall reservoir quality. To calculate the volume of shale of upper gas sand zone of Bokabil Formation from gamma ray log, firstly the gamma ray index has been calculated by using the following equation of Schlumberger^60^.

1 $${\text{I}}_{{{\text{GR}}}} = \, \left( {{\text{GR}}_{{{\text{log}}}} {-}{\text{ GR}}_{{{\text{min}}}} } \right) \, / \, \left( {{\text{GR}}_{{{\text{max}}}} {-}{\text{ GR}}_{{{\text{min}}}} } \right)$$

where, I_GR_ = gamma ray index, GR_log_ = gamma ray log reading of the formation, GR_max_ = maximum gamma ray log reading at shale base line, GR_min_ = minimum gamma ray log reading at sand base line.

The gamma ray logs of 4 different locations are de-spiked and normalized, then the highest range of gamma values considered shale base line and lowest range of values considered sand base line.

The I_GR_ is a linear responses of gamma ray which is empirically correlated to the fraction or percent of shale volume, where V_sh_ = I_GR_ and it provides upper limit of shale volume of the formation^61^. The studied Bokabil Formation of Surma basin in Bangladesh is of Mio-Pliocene age, thus the function of Larionov model for tertiary clastic rocks has been used to calculate more accurate fraction or percent of volume of shale of the formation by using the following formula^62,63^.

2 $${\text{V}}_{{{\text{sh}}}} = \, 0.0{83 }* \, \left( {{2}^{{({3}.{7 }*{\text{ IGR}})}} {-}{ 1}} \right)$$

The volume of sand calculated by the following relation,

3 $${\text{V}}_{{{\text{qurtz}}}} = { 1 } - {\text{ V}}_{{{\text{sh}}}}$$

where, V_qurtz_ = fraction of quartz volume, V_sh_ = fraction of shale volume.

#### Porosity calculation

The total porosity has been calculated from the combination of density porosity and neutron porosity by using the following equations depending on the presence or absence of gas in the formation^64,65^. First, the density porosity has been calculated from density log by using the following formula.

4 $$\Phi_{{\text{D}}} = \, \left( {\uprho_{{{\text{ma}}}} {-} \, \uprho_{{\text{b}}} } \right) \, / \, \left( {\uprho_{{{\text{ma}}}} {-} \, \uprho_{{\text{f}}} } \right)$$

where, Φ_D_ = fraction or percent of density porosity of the formation, ρ_ma_ = matrix density of the formation, for pure quartz sand 2.65 g/cm^3^, ρ_b_ = bulk density of the formation from log, ρ_f_ = fluid density, usually ranges from 1 to 1.1, but due to gas effect it becomes < 1 which results high porosity.

For gas bearing zone,

5 $$\Phi_{{\text{T}}} = \, \left( {\Phi_{{\text{D}}} + \, \Phi_{{\text{N}}} } \right) \, /{ 2}$$

For absence of gas in the formation,

6 $$\Phi_{{\text{T}}} = \sqrt {\frac{{\Phi_{D}^{2} + \Phi_{N}^{2} )}}{2}}$$

where, Φ_T_ = total porosity or accurate porosity of the formation, Φ_N_ = fraction or percent of neutron porosity of the formation.

The effective porosity is calculated by the following equation^66,67^.

7 $$\Phi_{{\text{e}}} = \, \Phi_{{\text{T}}} * \, \left( {{1 }{-}{\text{ V}}_{{{\text{sh}}}} } \right)$$

In places with bad boreholes, sonic porosity was used instead of density porosity due to its high depth of penetration. Sonic porosity was calculated from compressional sonic log by using the following equation^68^.

8 $$\Phi_{{\text{S}}} = \, \left( {\Delta {\text{t}}_{{{\text{log}}}} {-} \, \Delta {\text{t}}_{{,{\text{ma}}}} } \right) \, / \, \left( {\Delta {\text{t}}_{{\text{f}}} {-} \, \Delta {\text{t}}_{{{\text{ma}}}} } \right)$$

where, Φ_S_ = fraction or percent of sonic porosity of the formation, Δt_log_ = interval transit time from log, (μs/ft), Δt_ma_ = interval transit of the matrix of the formation, for sandstone 54–56 (μs/ft), Δt_f_ = interval transit time of fluids in the well bore, for mud filtrate 185–190 (μs/ft).

#### Water saturation

Water saturation was calculated by using Indonesian model, the following equation has been used^69^.

9 $$\text{Sw }= {[{\{{(\frac{{V}_{sh}^{2-{V}_{sh}}}{{R}_{sh}})}^{1/2} +{(\frac{{\Phi }_{e}^{m}}{{R}_{w}})}^{1/2}\}}^{2}]}^{-1/\text{n}}$$

where, S_w_ = water saturation, Φ_e_ = effective porosity, R_sh_ = resistivity of shale, V_sh_ = volume of shale, m = cementation coefficient, n = saturation capacity, R_w_ = formation water resistivity.

#### Permeability

Permeability estimation of a reservoir by using well log is one of the useful methods when core data are unavailable. The permeability has been calculated with the help of Timur empirical model based on porosity and irreducible water saturation by using the following equation^70^.

10 $$\text{K }= 0.136 * \frac{{\Phi }^{4.4}}{{S}_{wir}^{2}}$$

where, K = permeability of the formation in millidarcies (mD), Φ = total porosity, S_wir_ = Irreducible water saturation.

The irreducible water saturation has been calculated by the following equation.

11 $${\text{S}}_{\text{wir}} =\frac{\Phi * {\text{S}}_{\text{w}}}{{\Phi }_{\text{e}}}$$

where, S_w_ = water saturation of the formation, Φ_e_ = effective porosity of the formation.

## Results

### The Bokabil Formation

**Figure 3:**
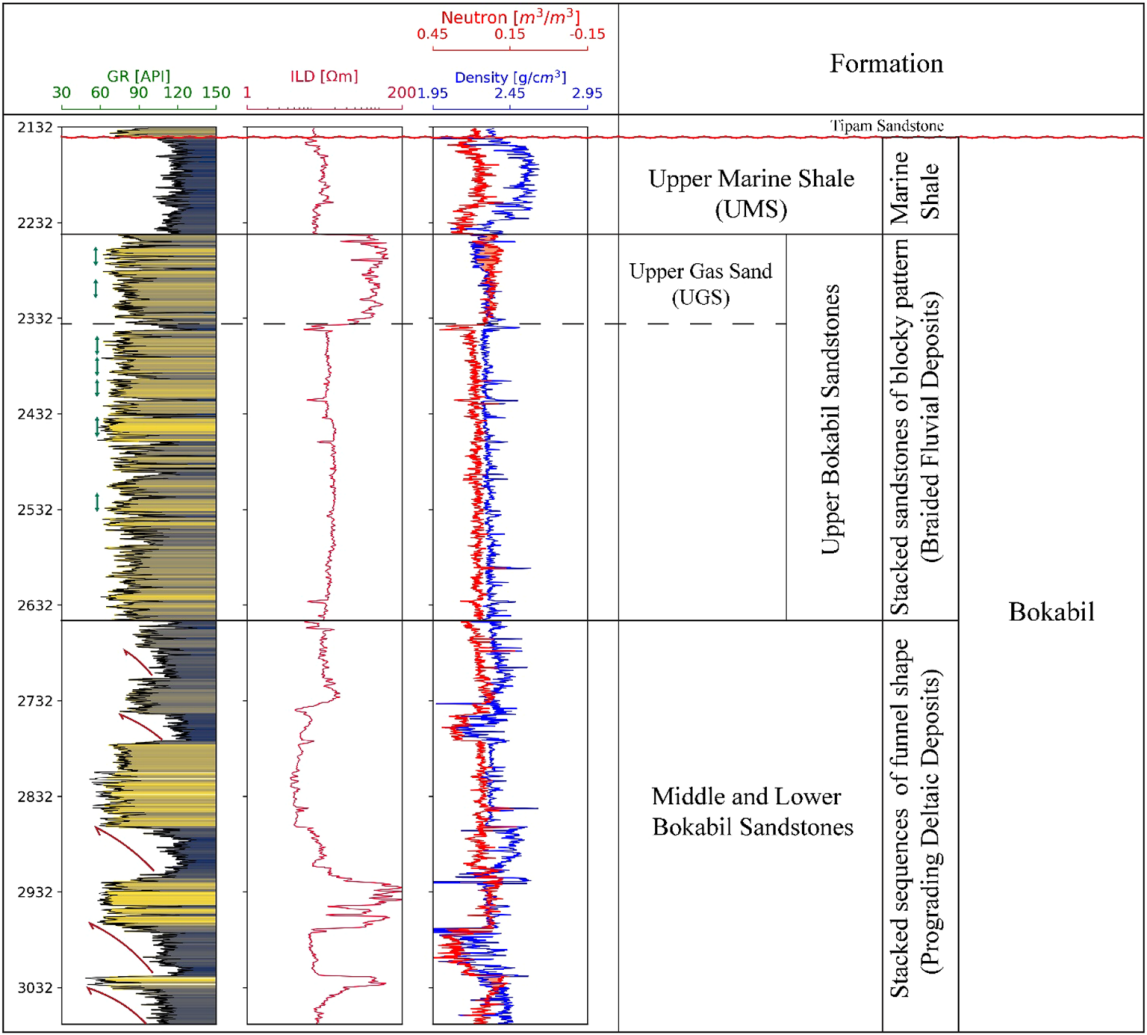
Wireline logs of Kailashtila well showing the most part of the Bokabil Formation. Uppermost part of the Bokabil Formation is known as the Upper Marine Shale followed by the Upper Bokabil Sandstones represented by the blocky sandstones (green color) and finally the Middle and Lower Bokabil Sandstones indicated by the coarsening upward cycles (red color).

The Bokabil Formation is around 1500 m thick in the Surma Basin^71,72^. Outcrop and subsurface data analysis suggest that there are significant variations in the rock types of this formation. The top most part of the formation is a thick shaly unit, popularly known as the Upper Marine Shale (UMS) (Fig. 3). This represents the last marine transgression and is acting as the regional seal in the Bengal Basin^59,73^. It has an unconformable contact with the overlying Tipam Sandstone Formation.

**Figure 4:**
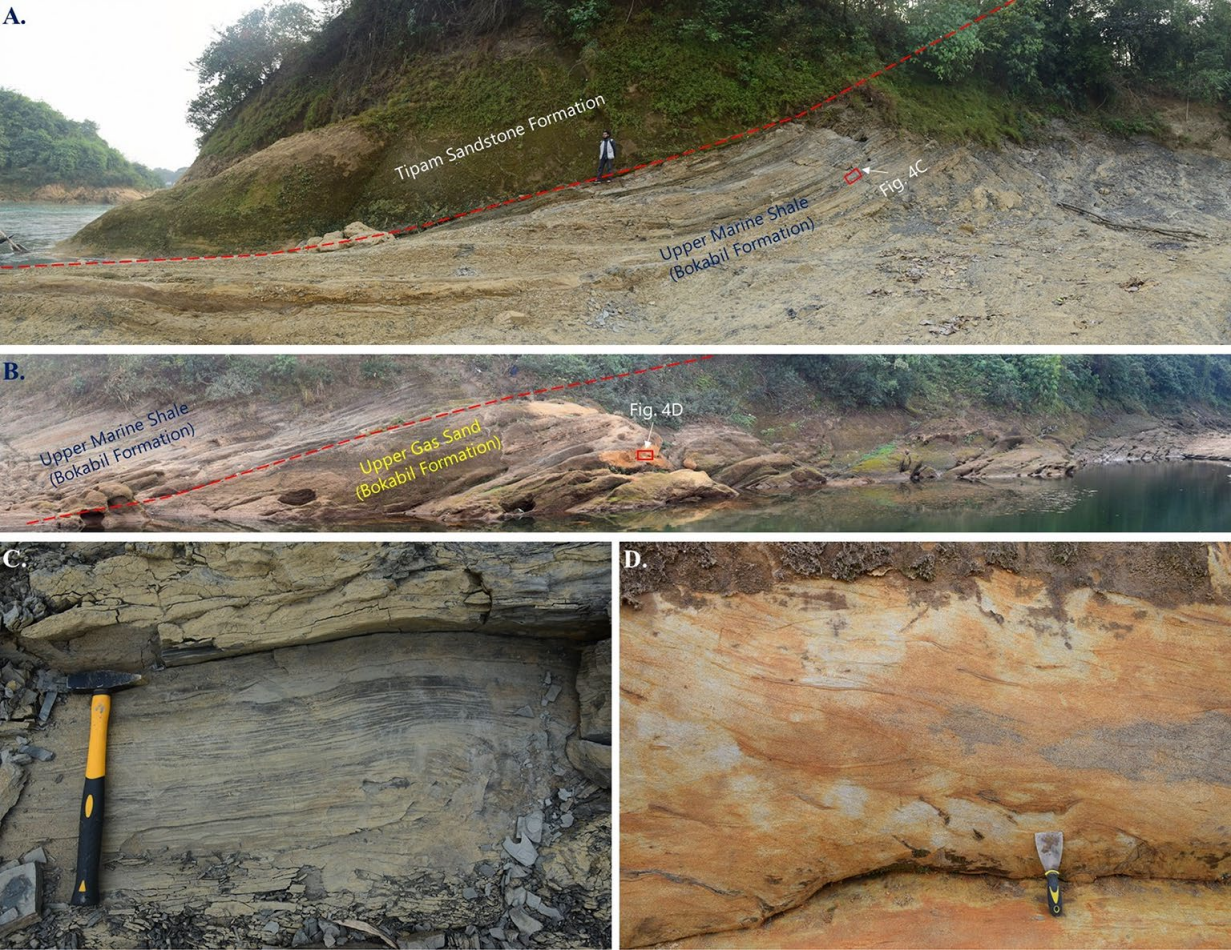
Outcrop photos of Bokabil Formation. (**A**) The contact between Bokabil Formation and the overlying Tipam Sandstone Formation, (**B**) contact between Upper Marine Shale (UMS) and Upper Gas Sand (UGS) within the Bokabil Formation, (**C**) Dark grey fissile shale is the characteristic lithology of Upper Marine Shale, (**D**) Yellowish brown, trough cross bedded sandstone is the dominant lithofacies in Upper Bokabil Sandstones.

In the outcrop this unit is characterized by dark gray to black fissile shale, and in the well logs, this unit shows high gamma ray value and high density. Following the Upper Marine Shale, a thick sequence of sandstone unit is identified (Fig. 4).

Log motif analysis shows the Upper Bokabil Sandstones are of blocky pattern and are separated by thin shale units of high gamma ray. Thickness of individual sand unit is in the range of few meters. Overall, this unit consisting with sandstones of blocky pattern, is around 350 m thick in the entire Surma Basin (Figs. 3, 9). Facies at the outcrops suggest this unit is of fluvial origin indicated by large cross beds and channel shaped sandbodies. These moderately consolidated sandstone units are collectively termed as the Upper Bokabil Sandstones^53^. Below these units are some coarsening upward cycles have been noticed. The outcrop equivalents of these units were not found in the study area. Based on the eletrofacies these are interpreted as the prograding deltaic deposits. Thickness of shale in these coarsening upward cycles decrease upward suggesting gradual shallowing of the basin^59,73^. The interpretation is consistent with the paleogeography and tectonic evolution of the Bengal Basin^73^. As the contact between Bokabil Formation and the underlying formation has not been observed in the studied wells, it is believed that significant portion of the Bokabil Formation has not been penetrated by the wells, hence these are termed as the Middle and Lower Bokabil Sandstones.

### Characterization of Bokabil Sandstone reservoir

#### Petrographic characterization

Fourteen (14) samples were prepared for the thin section and sieve analysis to study the texture, mineralogy, porosity and diagenetic history of the Upper Bokabil Sandstones.

##### Texture

**Figure 5:**
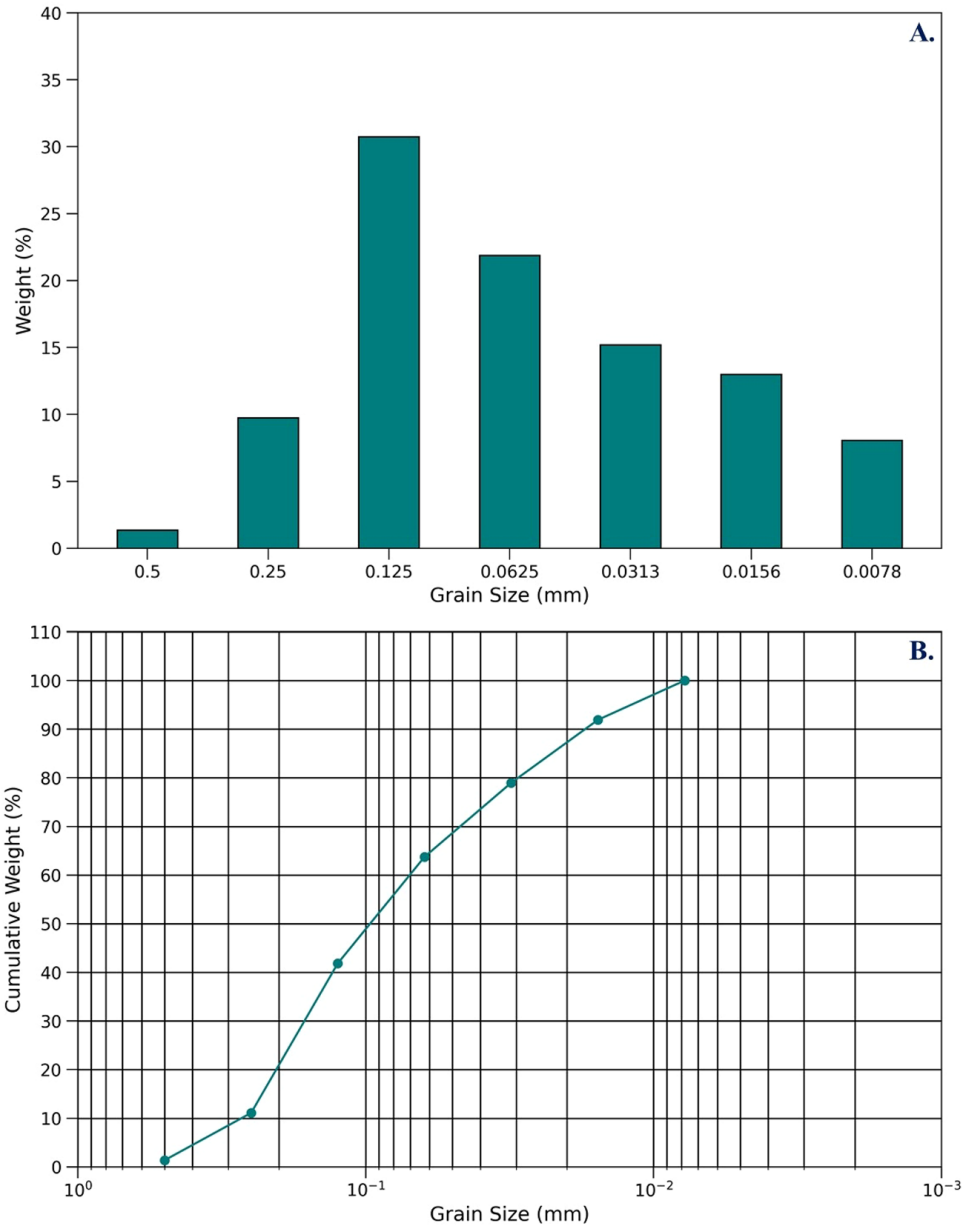
Grain size distribution of Upper Bokabil Sandstones showing unimodal distribution and the sandstones are predominantly fine grained.

The grain sizes are estimated on the basis of Udden–Wentworth scale^74,75^. The studied samples are fine to medium grained sand, moderately sorted, and sub-angular to sub-rounded in texture. The grain size sieve analysis including various statistical measurements suggest that the Upper Bokabil sandstones are unimodal and fine to medium grained in size, the modal class ranges between 0.125 and 0.0645 mm in grain size (Fig. 5) indicating the predominance of the fine sand. Various statistical parameters^76^ have been measured for the Upper Bokabil sandstones, where the graphic mean value is 2.23 which indicates fine sand, the graphic standard deviation or the sorting value is 0.78 indicates moderately sorted, the graphic skewness value is 0.95 indicates positive skewed or fine skewed, and the graphic kurtosis value is 1.17 which indicates leptokurtic.

##### Mineralogy

Thin sections were analyzed using petrographic microscope to study the mineralogical composition of Upper Bokabil Sandstones. Most of the thin sections show that the upper Bokabil Sandstones are composed of quartz, feldspars, rock fragments, and mica. The clay minerals are present as matrix and void spaces are the porosity.

**Figure 6:**
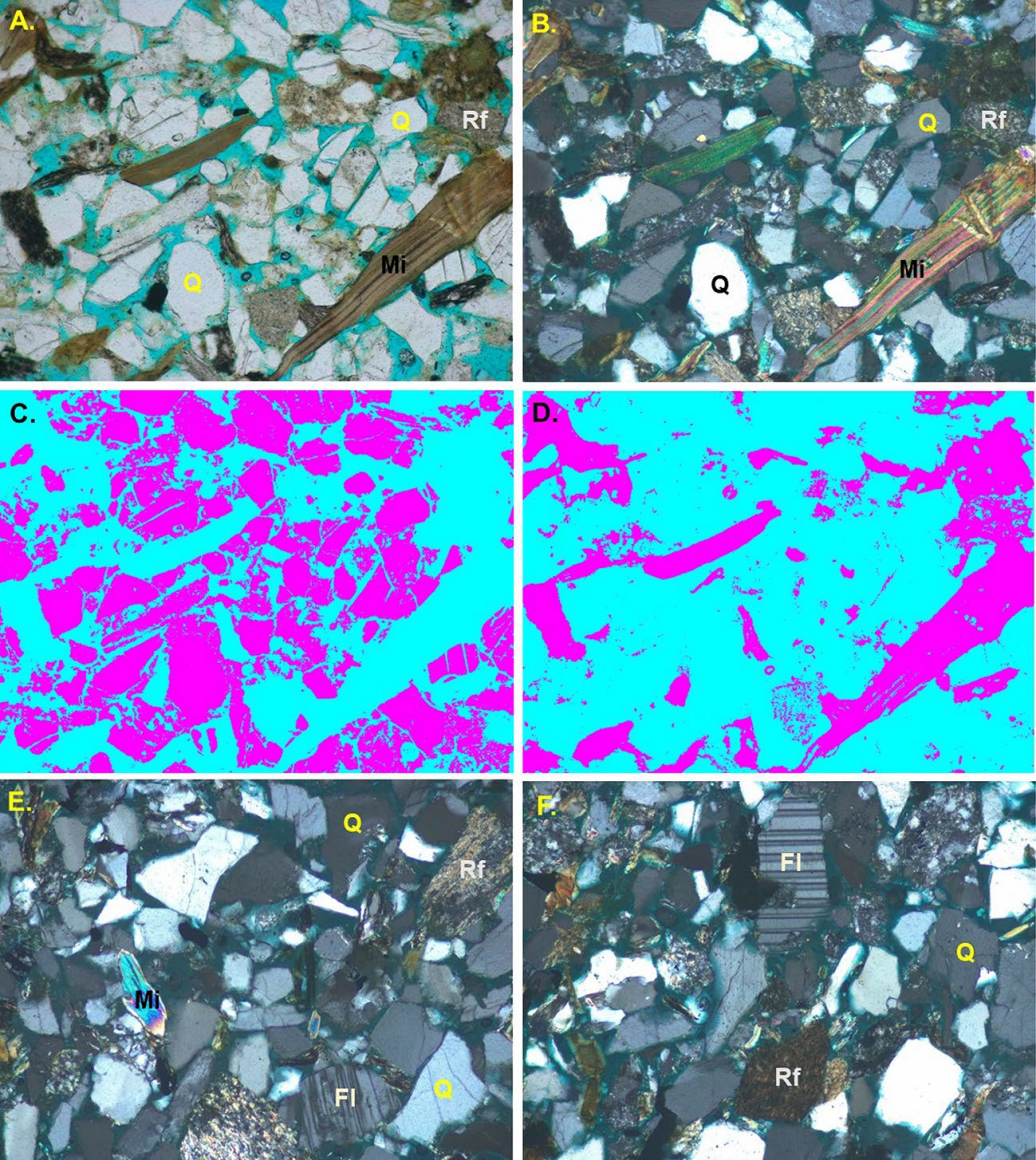
Photomicrographs of the representative thin section under plane (**A**) and cross (**B**) polarized light showing the constituent minerals (Q = Quartz, Mi = Mica, Fl = Feldspar, Rf = Rock Fragments). Quartz grains (**C**) and the Mica and Rock Fragments (**D**) separated using image segmentation techniques. Photomicrographs under crossed polarized light (**E**, **F**) showing Quartz, Feldspar, and Rock Fragments.

The unsupervised image segmentations using machine learning can effectively separate quartz (Fig. 6C) and mica (Fig. 6D) which helps in measuring amount of various minerals. Quartz is the most abundant mineral in the samples with a percentage of about 65–75 percent (Fig. 6A). Feldspars, mica and rock fragments comprises about 5–12 percent, 3–8 percent, and 2–4 percent, respectively (Fig. 6E,F).

##### Diagenesis

**Figure 7:**
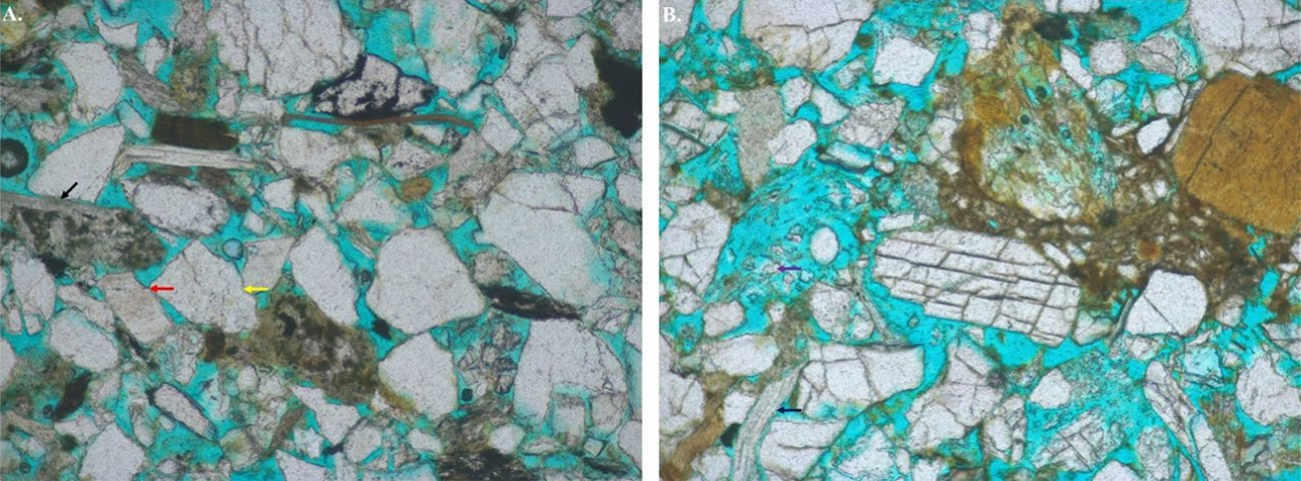
Photomicrographs under plane polarized light showing different type of diagenetic features such as line contact (blue arrow), point contact (red arrow), suture contact (yellow arrow) and dissolved grains (purple arrow).

Diagenesis plays a vital role in both enhancing and destroying reservoir quality. The dissolution of fragmental grains including changes in grain shape, contacts, deformation of the soft grains, generation of secondary porosity all are resulted due to physical, chemical and physicochemical changes, which all are involved in the stage of diagenesis of the Upper Bokabil Sandstones in the Surma Basin. Predominance of point and line contacts (Fig. 7) among grains suggest moderate compaction occurred at shallow depth. Some suture contacts and bending of elongated grains also been noticed. The secondary porosities are resulted from dissolution of structural grains. The feldspars are most susceptible for secondary porosity generation by the process of dissolution. In some grains dissolutions of feldspars have removed 70–80% of the grain.

##### Porosity

**Figure 8:**
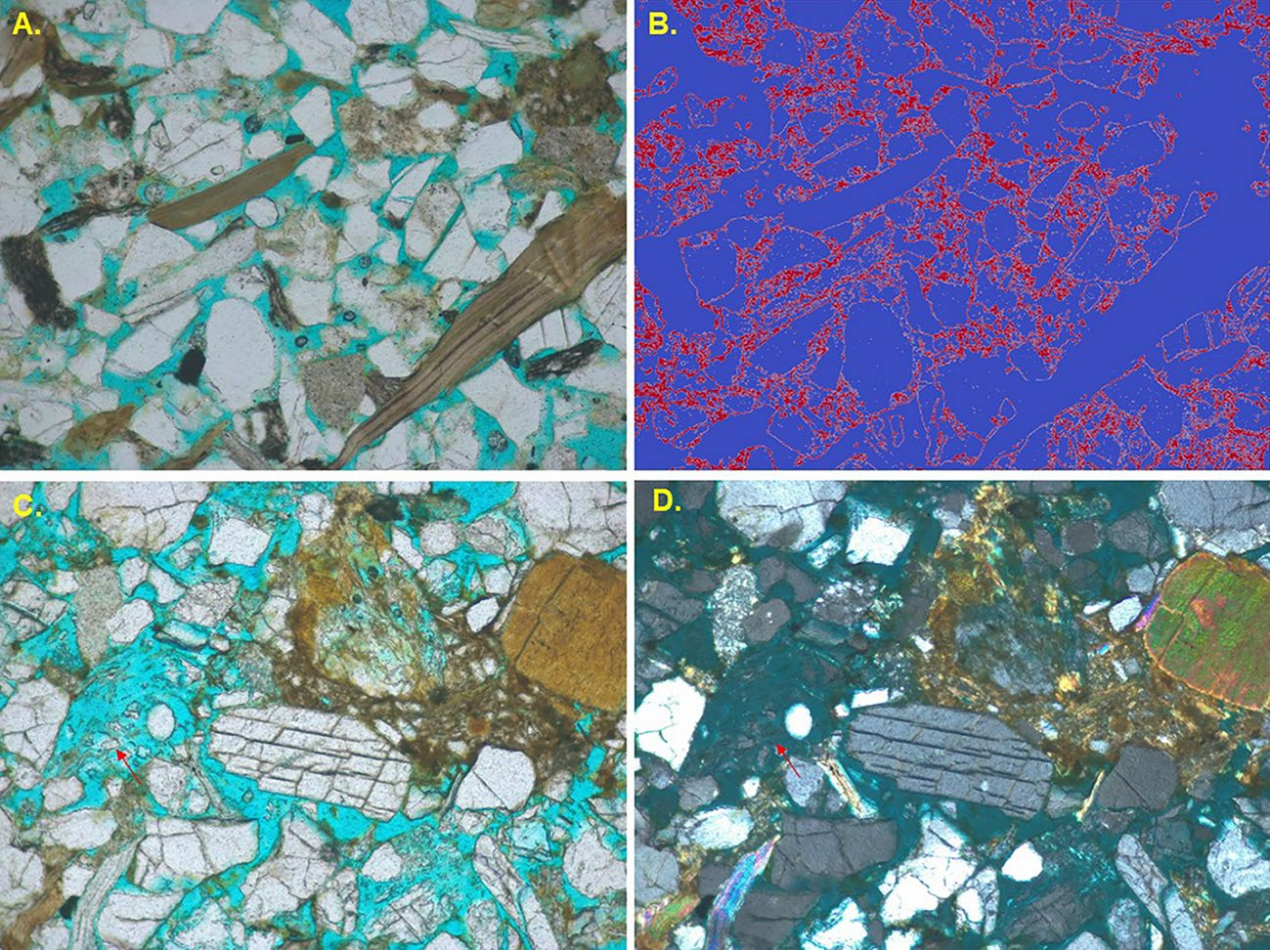
Photomicrograph under plane polarized light (**A**) showing the primary intergranular porosity (blue dye). Pore networks (red color) extracted using image segmentation technique showing good connectivity among pores (**B**). Photomicrographs showing the secondary porosity (red arrow) under plane cross polarized light (**C**, **D**).

The porosity of the samples was measured in thin section by point counting and area coverage. Porosity in the studied samples ranges from 18 to 26% (Fig. 8A) including mostly primary porosity and some secondary porosity. The secondary porosity mainly generated by dissolution of framework grains (Fig. 8C,D) after burial. The dissolutions took place within plagioclase feldspars than other framework grains which supporting the Goldich Weathering Series^77^. The petrographic analysis by using unsupervised image segmentation with OpenCV reveals a well connected pore network (Fig. 8B) within the thin section of Bokabil Sandstone.

#### Petrophysical analysis

**Figure 9:**
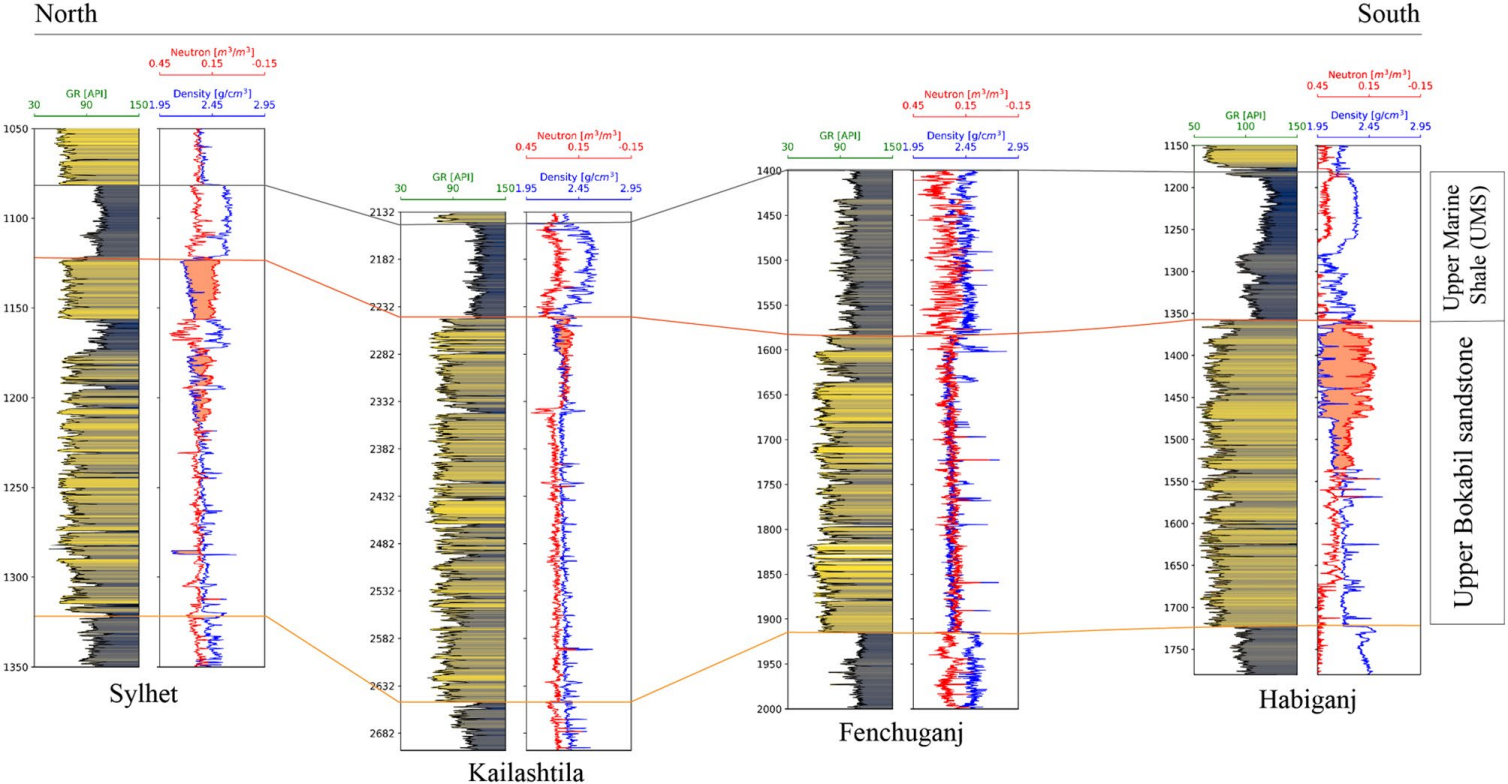
North–south correlation panel across the studied wells showing the variation in the thickness of Upper Marine Shale (UMS) and Upper Bokabil Sandstone. Thickness of UMS increases from north to south. There is no obvious trend in the Upper Bokabil Sandstone thickness but seem to have a uniform thickness throughout the basin.

The petrophysical analysis has been carried out to evaluate reservoir properties and eventually to estimate the storage capacity. The estimated petrophysical properties are volume of shale and their distribution, porosity, permeability, and water saturation. The Upper Bokabil Sandstones in the Surma Basin are observed at different depths at different wells including 1195–1590 m, 2245–2650 m, 1355–1720 m and 1580–1910 m in the Sylhet, Kailashtila, Habiganj and Fenchuganj structures (Fig. 9), respectively. The estimated net reservoir thickness of the Bokabil Formation is about 351 m, 353 m, 271, 332 m in the Sylhet, Kailashtila, Fenchuganj, and Habiganj structures (Fig. 9), respectively.

##### Volume of Shale

The volume of shale and distribution of shale and distribution of shale types including dispersed, laminated and structural can affect porosity, permeability and saturation as well as log responses of a reservoir^78^. The histogram of volume of shale reveals the distribution of volume of shale percentage in the reservoir sands of Bokabil Formation.

**Figure 10:**
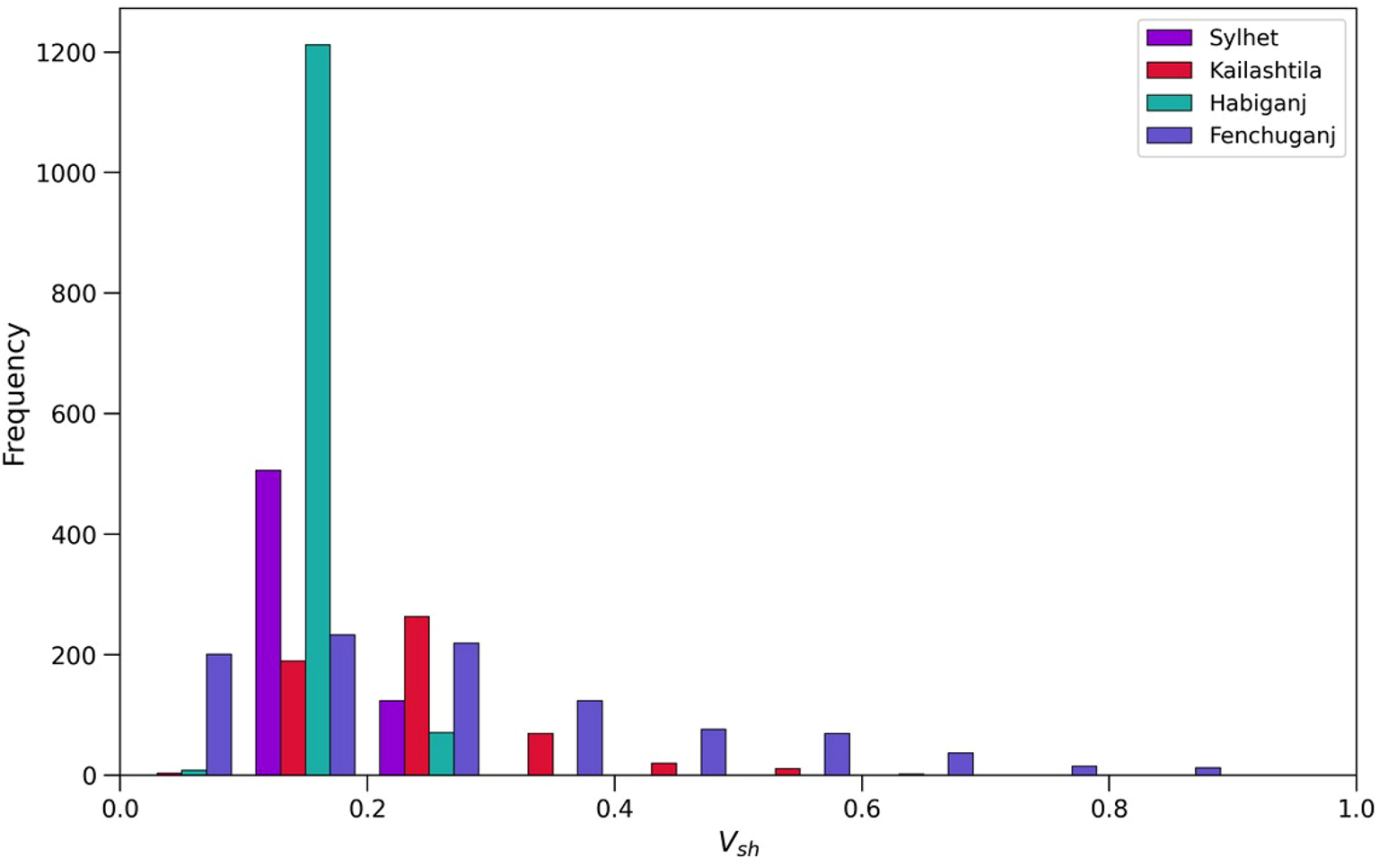
Histogram of the Vsh distribution in the Upper Bokabil Sandstone unit in four gas fields.

The log plots and the histogram for volume of shale (Fig. 10) of reservoir sands reveal that the average volume of shale in the Sylhet structure is about 17.7%. In the Kailashtila, Habiganj and Fenchuganj structures the average volume of shale are about 24%, 15%, and 27%, respectively.

##### Shale distribution

**Figure 11:**
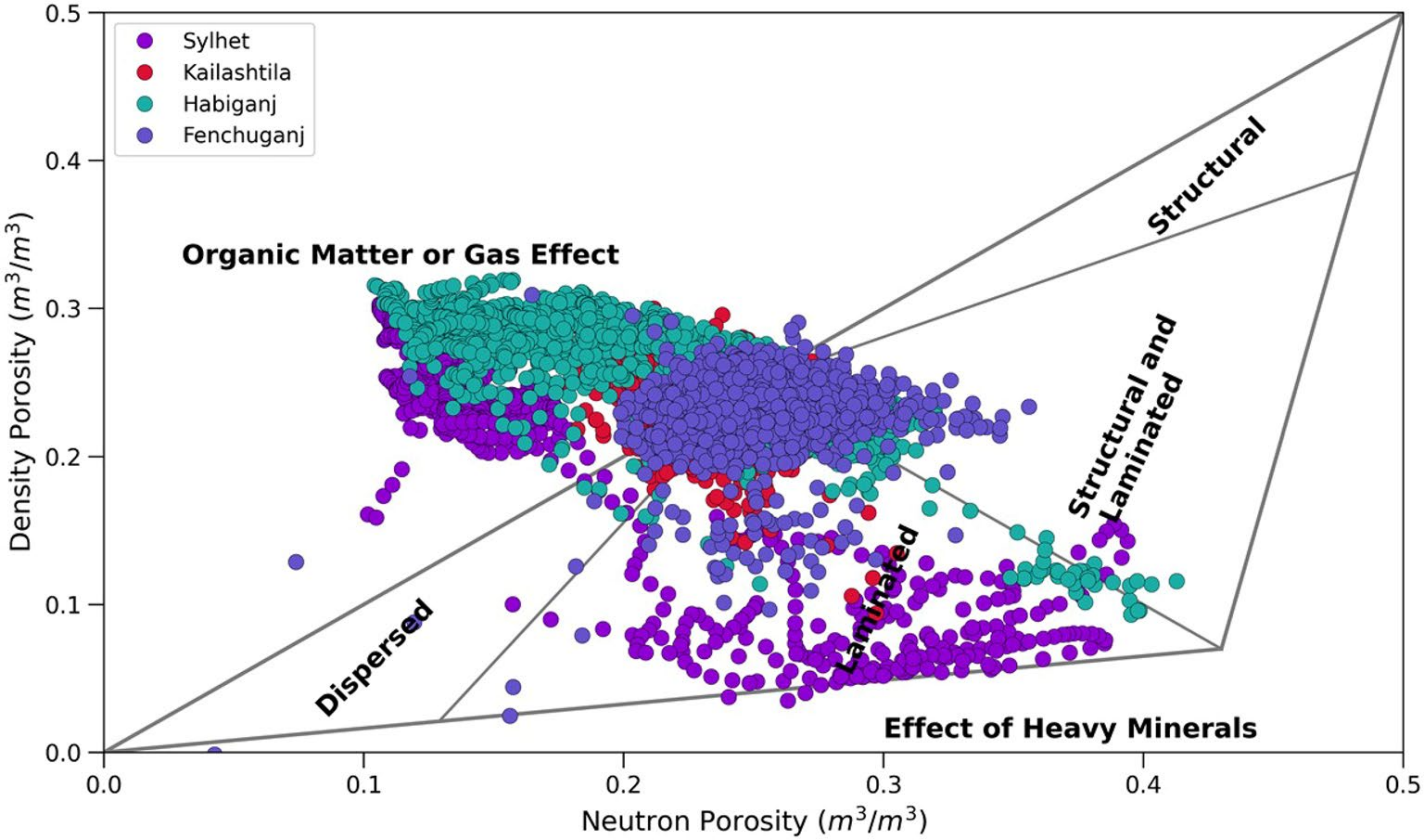
Crossplot showing the type of shale distribution in the Upper Bokabil Sandstone in four gas fields. Shales are mostly distributed in laminated fashion.

**Figure 12:**
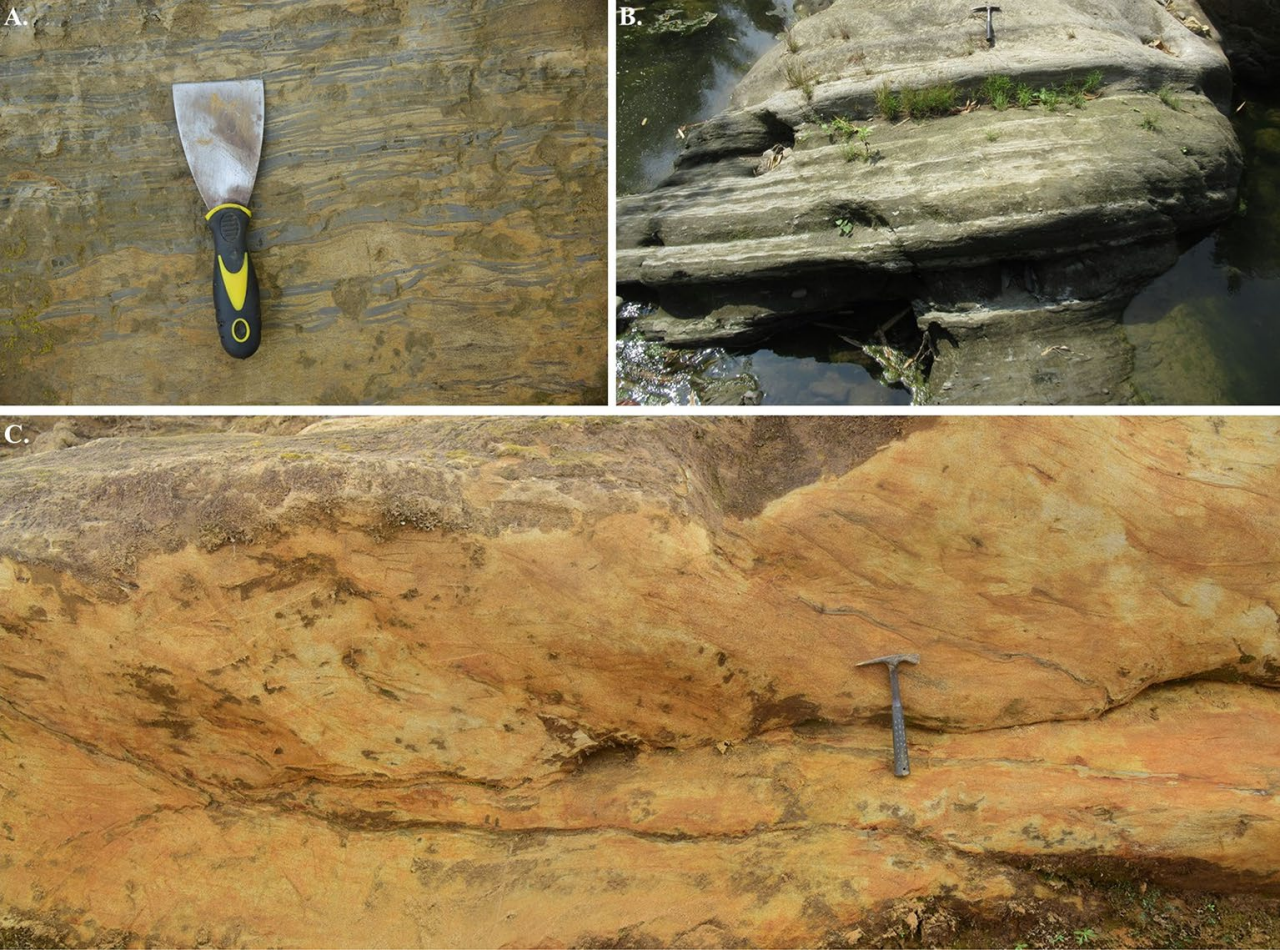
Outcrop facies showing the laminated shales alternated with sandstones (**A**) and siltstone (**B**). Clay also occurs as mud drapes along the forsets of the crossbedded facies (**C**).

Density porosity and Neutron porosity crossplot also known as Thomas Stieber cross plot^79^ was used to identify the shale distribution pattern within the Upper Bokabil Sandstone. The plot shows that most of the points of all four wells fall within the domain of laminated shale. Some are mixed structural and laminated shale (Fig. 11). There is hardly any shale that is dispersed within the sands. In the studied thin sections presence of structural shale could not identified. Observations in the outcrops show prevalence of shale facies alternated with sandstone and siltstone (Fig. 12). These also occur as mud drapes over the foresets in the planar and trough cross bedded sandstone facies.

##### Porosity

**Figure 13:**
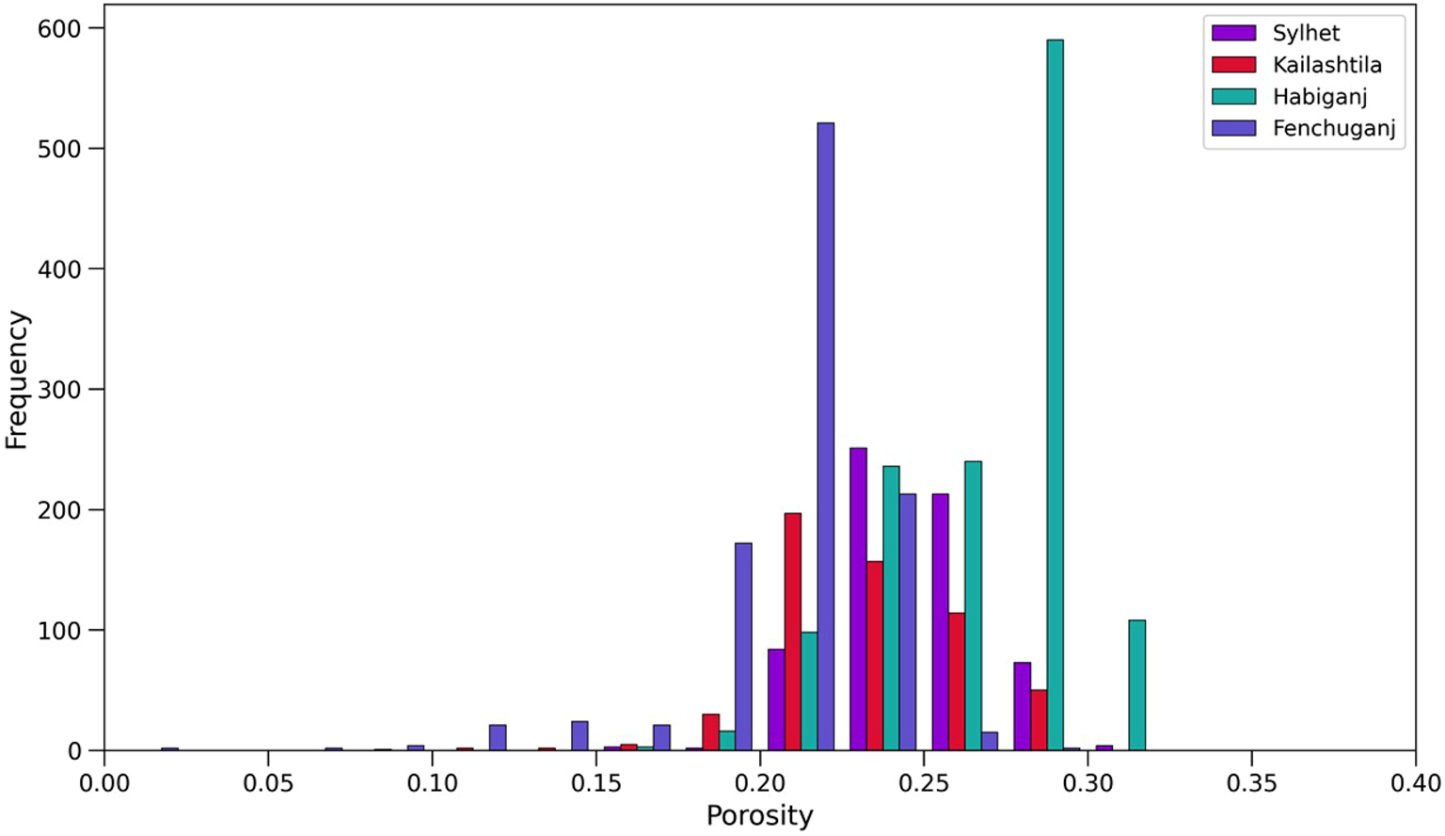
Histogram of the porosity distribution in the Upper Bokabil Sandstone unit in four gas fields.

**Figure 14:**
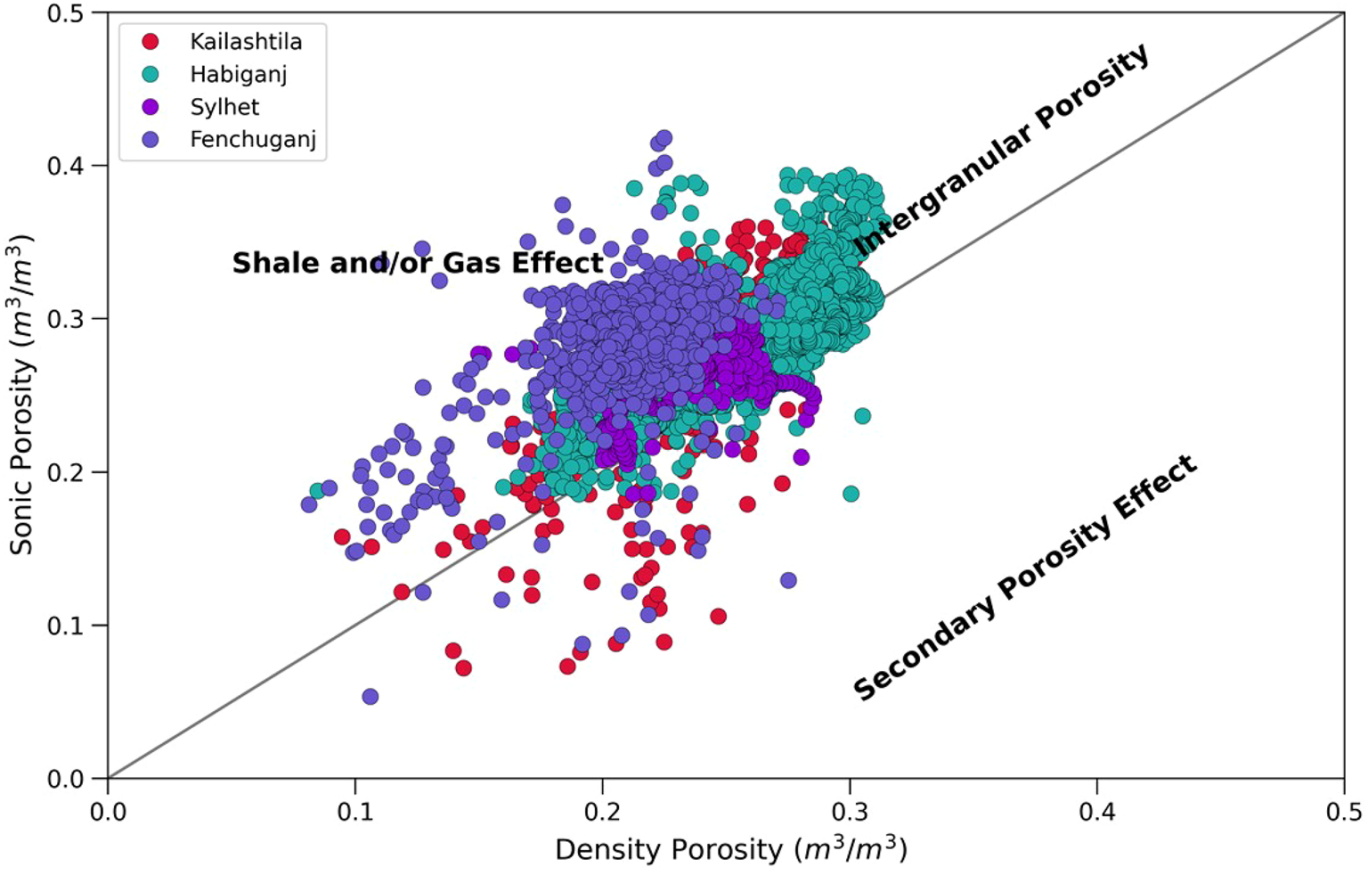
Crossplot showing the dominant porosity in the Upper Bokabil Sandstone unit is the primary porosity in all four gas fields.

The histogram of porosity (Fig. 13) for Sylhet, Kailashtila, Habiganj and Fenchuganj structures shows the distribution of porosity within the Upper Bokabil Sandstone. The average porosity is about 24.8%, 23.4%, 27.4% and 23.86% in the Sylhet, Kailashtila, Habiganj and Fenchuganj structures, respectively. The Upper Bokabil Sandstone in Kailashtila and Fenchuganj structures shows relatively lower porosity than Sylhet and Habiganj structures due to the position of reservoir sands at greater depth than Sylhet and Habiganj structures. The cross plot of sonic porosity against density porosity shows these are mostly primary intergranular porosity (Fig. 14). Relatively minor secondary porosities could be related to the dissolution of grains as seen in the thin sections (Fig. 8C,D).

##### Permeability

**Figure 15:**
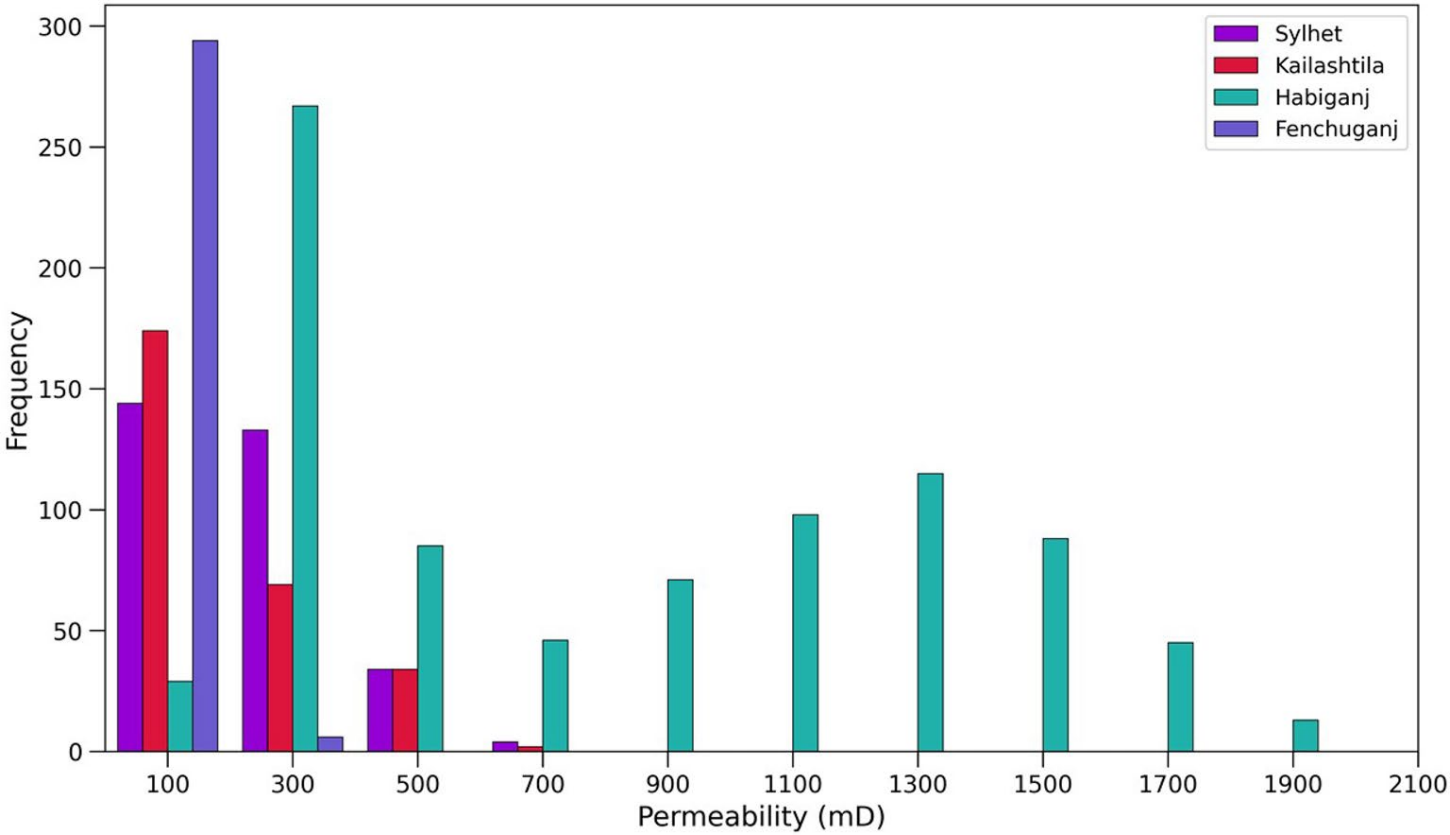
Histogram of the permeability distribution in the Upper Bokabil Sandstone unit in four gas fields.

The histogram of permeability (Fig. 15) of Upper Bokabil Sandstone shows the distribution of permeability in the Sylhet, Kailashtila, Habiganj and Fenchuganj structures. The average permeability is about 241 mD, 209 mD, 857 mD and 140 mD in Sylhet, Kailashtila, Habiganj and Fenchuganj structures, respectively. The Kailashtila and Fenchuganj structures show lower permeability than Sylhet and Habiganj structures due to lower porosity, position at greater depth and higher laminated shale distribution (Fig. 11). The Sylhet structure showing lower permeability than Habiganj structure due to lower porosity and presence of higher proportion of laminated shale.

##### Water saturation

**Figure 16:**
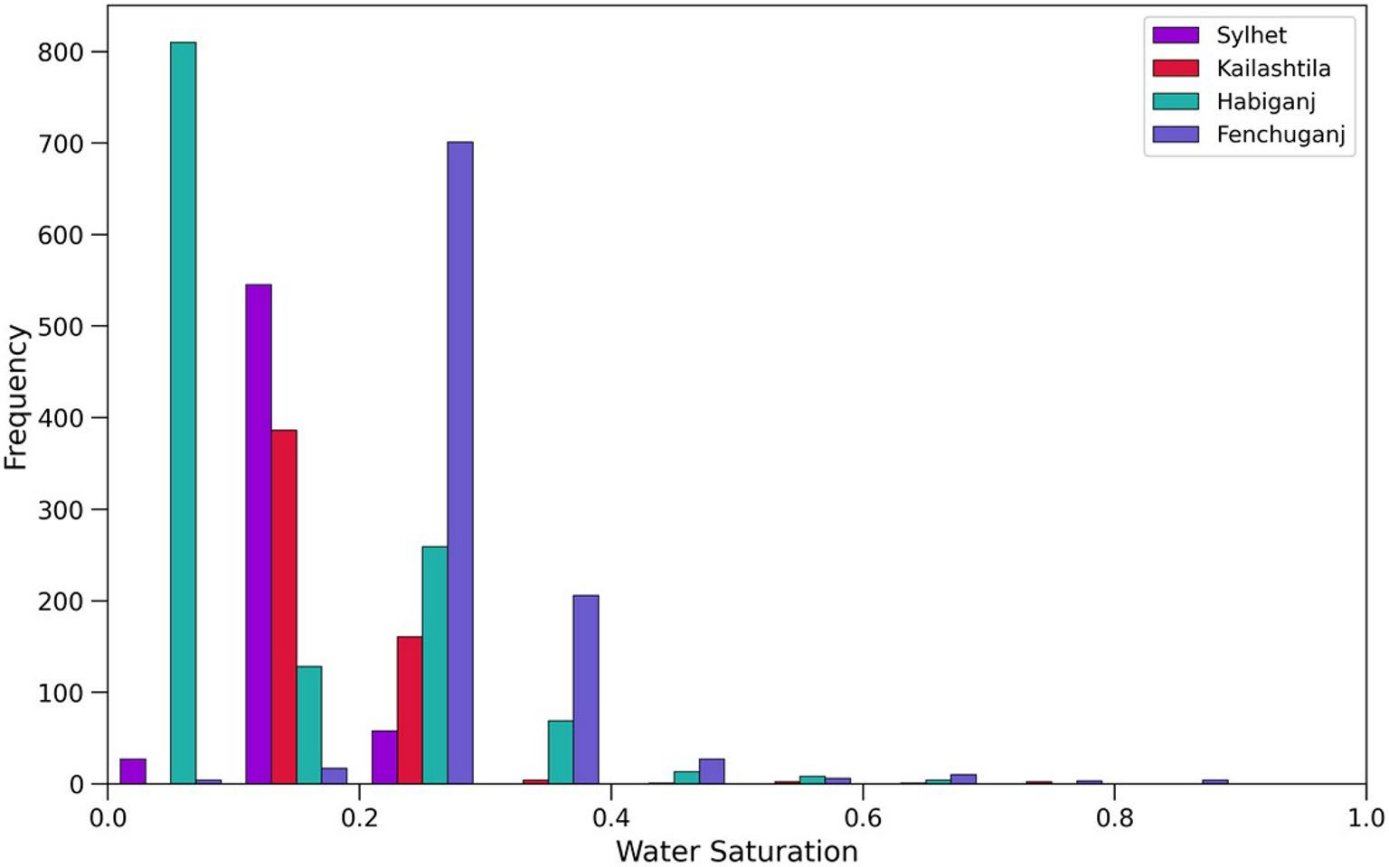
Histogram of the water saturation distribution in the Upper Bokabil Sandstone unit in four gas fields.

The histogram of water saturation (Fig. 16) of Upper Bokabil Sandstone shows the distribution of water saturation in Sylhet, Kailashtila, Habiganj and Fenchuganj structures. The average water saturation is about 14.3%, 18.7%, 15.7% and 21% for the Sylhet, Kailashtila, Habiganj and Fenchuganj structures, respectively.

The average petrophysical properties of Upper Bokabil Sandstone in all four gas fields have been summarized in Table 2.

**Table 2:** Summary of the petrophysical properties of Upper Bokabil sandstones in the four gas fields.

Well name	Thickness (m)					Porosity (%)		Shale (%)	Fluids (%)	Permeability (mD)
	Top	Bottom	GT	NST	NRT	Φ_T_	Φ_e_	V_sh_	S_w_	K
Sylhet	1195	1590	395	360	351	24.8	20.4	17.6	14.3	241
Kailashtila	2245	2650	405	365	353	23.4	17.8	24	18.7	209
Habiganj	1355	1720	365	338	332	27.4	23.3	15	15.7	857
Fenchuganj	1580	1910	330	285	271	23.86	17.42	27	21	140

Here, multiple cut-offs applied for the determination of net pay. The used cut-offs are Φ_T_ ≤ 9%, V_sh_ ≥ 60%, and S_w_ ≥ 60%. Where, Φ_T_, Φ_e_, V_sh_, K and S_w_ have been denoted as Total Porosity, Effective Porosity, Volume of Shale, Permeability and Water Saturation, respectively. GT, NST, and NRT indicate Gross Thickness, Net Sand Thickness, and Net Reservoir Thickness, respectively.

##### Flow barriers and the Kv/Kh

The outcrop of Upper Bokabil sandstone exhibits various permeability barriers (Fig. 12) that might play a crucial role in controlling vertical permeability. These barriers are present at different scales, ranging from the smallest to the largest. The smallest-scale barriers are mud laminae that alternate with sand laminae (Fig. 12). In addition, mud drapes along the foresets of cross-bedded sandstone units can also impede flow. At the largest scale, the most significant barriers are floodplain mudstones, which alternate with channel deposits^53^. These barriers are laterally discontinuous. Such flow barriers can influence the injectivity and connectivity during the CO_2_ sequestration process^80–82^. Their effect on the vertical permeability has been described by many authors^83–85^. Here, the impact of discontinuous shale/mudstone on vertical permeability of the Bokabil Sandstone has been evaluated. The first-order influence of the discontinuous mudstones on vertical permeability has been evaluated by applying the equation developed by Begg and King^83^ to account for the effect of stochastically distributed, laterally discontinuous impermeable barriers (Fig. 12).

12 $$Kvs=K\times \frac{1-Fr}{({1+s\times \frac{l}{2})}^{2}}$$

where, Kvs is the effective vertical permeability, K is the effective vertical permeability in the absence of barriers, Fr is the fraction of barrier within the unit, s is the barrier density, and l is the mean barrier length. Considering Kv = Kh in the case of homogeneous sandstone, this equation can be rearranged to

13 $$Kvs/Kh=\frac{1-Fr}{({1+s\times \frac{l}{2})}^{2}}$$

Based on outcrop analysis (Fig. 12), s = 0.5 m^−1^, Fr = 0.15, and l = 8 m. By inputting these values to Eq. (13), the Kvs/Kh ratio equals 0.094, which suggests these mud layers have a significant impact on effective vertical permeability.

### Characterization of the Bokabil Caprock

**Figure 17:**
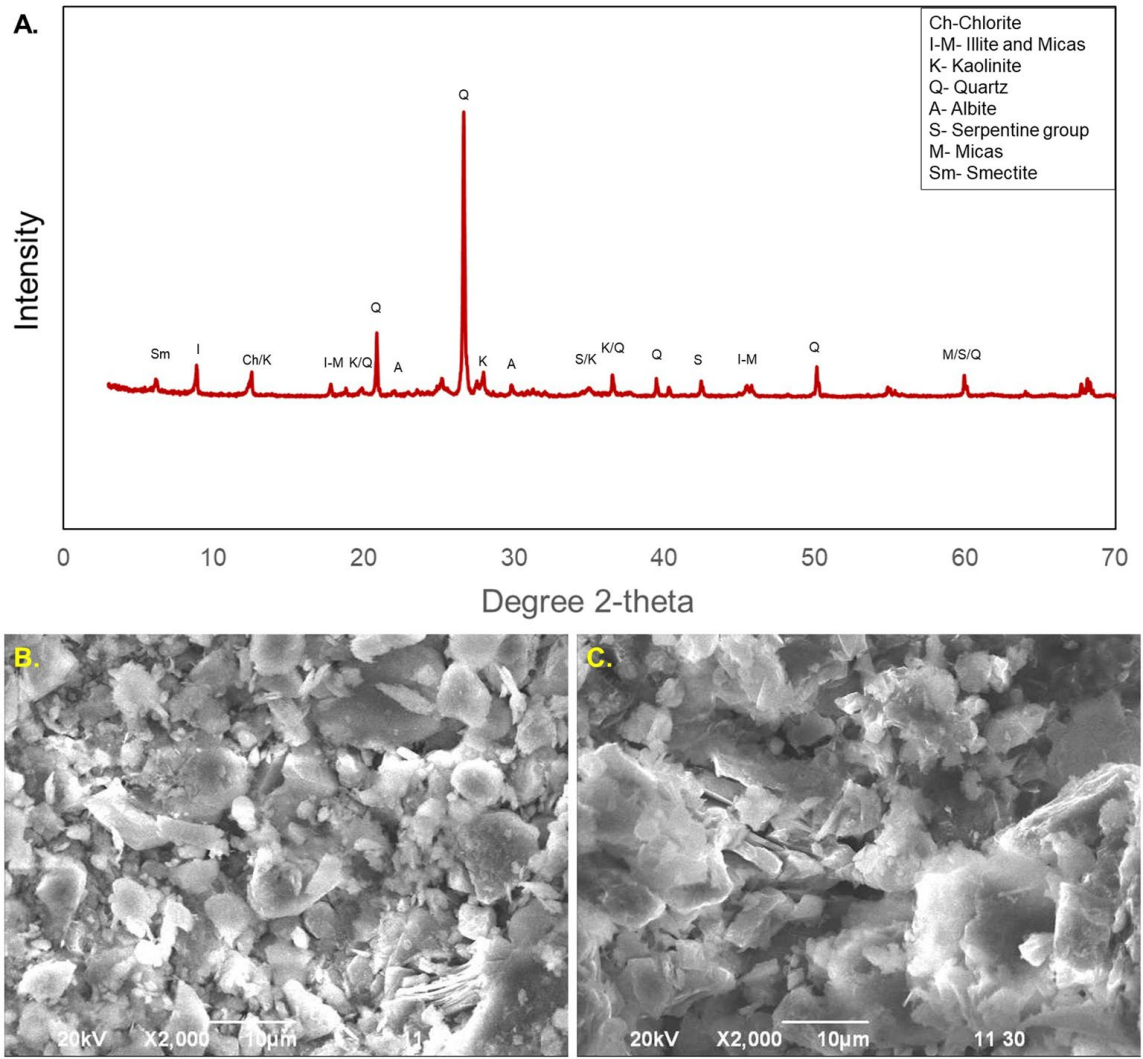
X-ray diffraction of UMS showing the constituent minerals (**A**) and the scanning electron microscope images showing the prevalence of flaky clay minerals (**B**, **C**).

The Upper Marine Shale unit of Mio-Pliocene age marked as Transgressive System Tract (TST) overlying the Bokabil reservoir units by 80–350 m thick impermeable rock unit as a cap rock succession^59,73^. The XRD analysis (Fig. 17A) reveals that the cap rock unit is mineralogically composed of quartz, albite, mica, illite, carbonate, kaolinite, smectite, chlorite, serpentine and a very few contaminations (Table 3). The percentages of abundant minerals quartz, feldspars, mica, illite and kaolinite in UMS are approximately 34%, 8%, 20%, 14% and 18%, respectively.

**Table 3:** Constituent minerals of the Upper Marine shale and their percentage.

Sand (> 63 μm)	Silt (2–63 μm)	Clay (< 2 μm)	Mineral (%)								TOC (%)
			Qtz	Felds	Mica	Illite	Kaol	Smect	Chlor	Mont	
0–8%	36–54%	45–55%	34	8	20	14	18	2	2	1	0.7–1.2

SEM images show the abundance of flaky minerals (Fig. 17B,C) which are identified as the clay minerals. The SEM images also reveal the nano type pore with very small diameters, in the range of 10–40 nm (Fig. 17B,C). This very small pore throat diameter results very high capillary entry pressure approximately 5–20 MPa based on the Washburn equation, where considering the interfacial tension for CO_2_–water and CO_2_–gas–water multiphase system is about 0.05 N/m based on the pendant drop technique and water wet condition for the contact angle (θ = 0°) between solid and fluid^86^.

### Effective storage capacity of Bokabil reservoirs

Effective storage capacity has been calculated to estimate total volume of CO_2_ that can be injected. The reservoir temperature and thermal gradient data (Table 4) are collected from^87^. The structural area (Table 4) calculated from the top to spill point on a seismic section. The average density of CO_2_ (Table 4) is estimated based on the depth-density cross plot^88^.

**Table 4:** Parameters used for the volumetric calculations.

Geological data	Field			
	Sylhet	Kailashtila	Habiganj	Fenchuganj
Area of the structure (km^2^)	39	43	74	87
Caprock thickness (m)	40	94	190	180
Reservoir thermal gradient (°C/100 m)	3.15	2.65	3.2	2.87
Reservoir temperature (°C)	37.64	59.49	43.36	45.34
Average density of CO_2_ at reservoir condition (kg/m^3^)	680	700	685	690
Compressional sonic velocity (m/s)	2250–2900	2500–3200	1900–2700	2450–2700

To calculate the theoretical CO_2_ storage capacity of the Sylhet, Kailashtila, Habiganj and Fenchuganj structures, the CSLF (Carbon Sequestration Leadership Forum) method has been used in this research paper^89^. The upper portion of the Upper Bokabil Sandstone is a gas reservoir while the lower portion a saline aquifer (Fig. 9). Therefore, Eq. (14) has been used for the brine aquifer portion of the reservoir and Eq. (15) has been used for the gas reservoir to calculate the total volume of CO_2_ can be stored in the studied geological structures using the CSLF method,

The equation for the brine aquifer:

14 $${\text{M}}_{{{\text{CSLF }}({\text{brine}})}} = \, \uprho {\text{CO}}_{{2}} .{\text{A}}.{\text{ h}}. \, \Phi . \, \left( {{1 }{-}{\text{ S}}_{{{\text{wirr}}}} } \right)$$

The equation for the gas reservoir:

15 $${\text{M}}_{{{\text{CSLF }}({\text{gas}})}} = \, \uprho {\text{CO}}_{{2}} .\left[ {{\text{R}}_{{\text{f}}} .{\text{ A}}.{\text{ h}}. \, \Phi . \, \left( {{1 }{-}{\text{ S}}_{{\text{w}}} } \right)} \right]$$

where, M_CSLF (brine)_ = CO_2_ storage capacity (kg) for the brine aquifer, M_CSLF (gas)_ = CO_2_ storage capacity (kg) for the gas reservoir, ρCO_2_ = average density of CO_2_ under reservoir conditions (kg/m^3^), A = area of the structure (m^2^), h = average thickness of the structure (m), Φ = average porosity of the structure, S_wirr_ = irreducible water saturation, the average irreducible water saturation assumed 25% for all structures, S_w_ = water saturation using from Table 2, R_f_ = recovery factor (average recovery factor about 85% based on production data of Petrobangla).

To estimate the theoretical storage capacity, the required values of variables for Eqs. (14) and (15) are taken from Tables 2 and 4 through log analyses. Three different cases have been considered for volumetric calculations. Petrophysical properties were varied by 2 standard deviations from the mean for the minimum and maximum cases. The standard deviations for porosity in the Sylhet, Kailashtila, Habiganj, and Fenchuganj structures are 0.022, 0.028, 0.029, and 0.0277, respectively. For water saturation, the standard deviations are 0.04, 0.07, 0.11, and 0.12 for the same structures, respectively. The areal coverage was also varied as a function of the height of the structural closure. Thickness was varied based on the observations in the studied wells.

**Figure 18:**
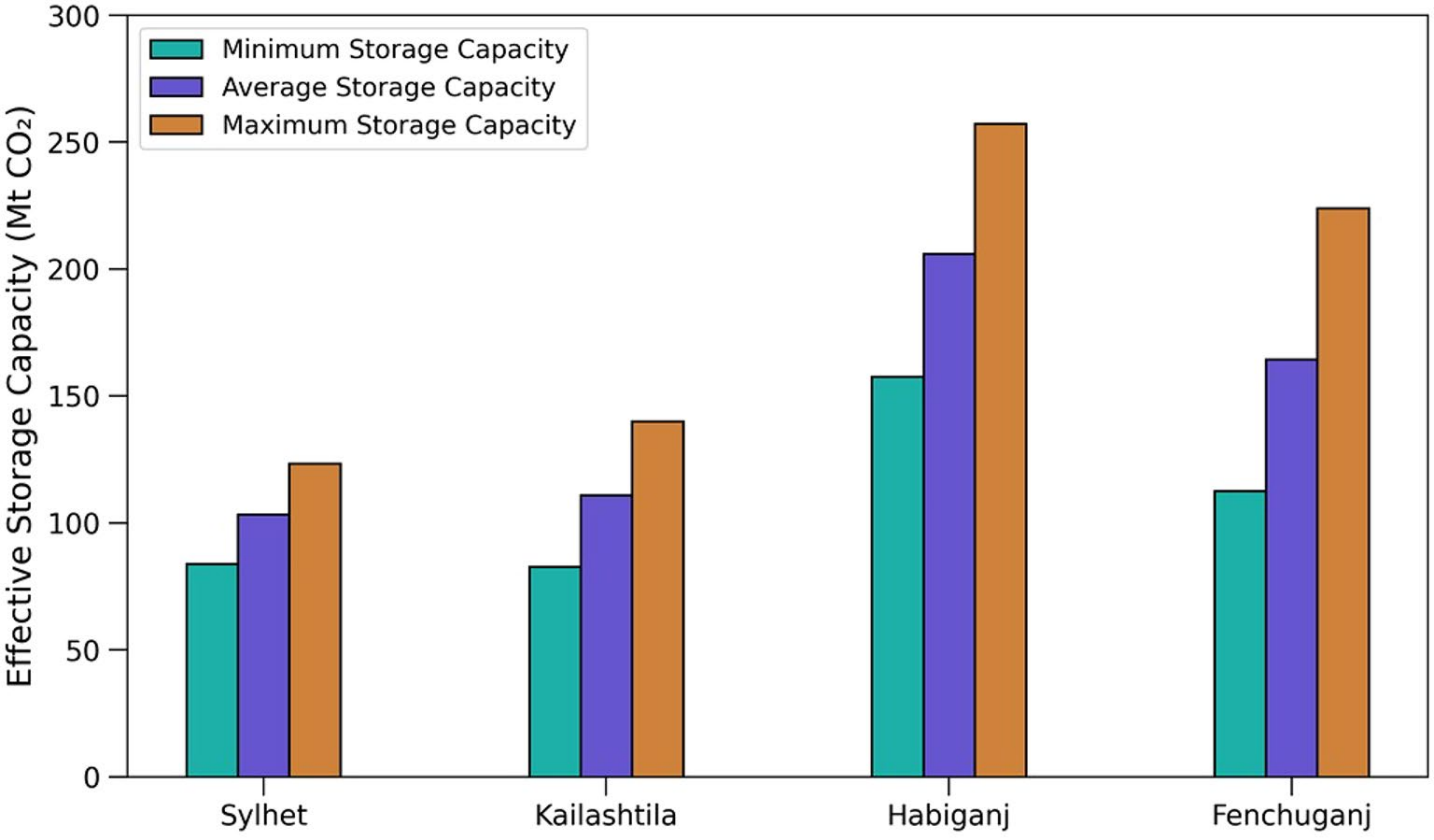
Effective storage capacity in all four gas fields. Minimum, average, and maximum effective storage capacity have been calculated from theoretical storage capacity by using 6% cut-off.

The storage efficiency for CO_2_ storage defines the ratio of substantive amount of CO_2_ stored to the available theoratical storage, that reveals the actual effective storage capacity^90^. By using multiphase flow theory and empirical site data, Ringrose et al. suggested the the actual effective storage capacity for CO_2_ injection is less than 6% of the theoratical storage capacity^91^. In case of Sleipner CO_2_ storage project, the storage efficiency was approximately 5.2%^92^. Therfore, 6% cut-off has been used to the theoretical storage capacity to estimate the actual effective storage capacity for CO_2_ injection (Fig. 18).

## Discussion

This study aimed at characterizing the Upper Bokabil Sandstones as a potential CO_2_ storage unit and the Upper Marine Shale as a regional seal by integrating subsurface data from four gas fields along with their outcrop analogues. Thin section petrography, SEM, XRD analysis, petrophysical analysis were performed to estimate the reservoir and seal characteristics.

The Upper Marine Shale (UMS), lying above the Upper Bokabil Sandstone is regionally extensive (Fig. 9) and effectively trapped the hydrocarbons below it in the Bengal Basin^25,72,93^. High percentage of quartz in the UMS (Fig. 17A) suggest its high mechanical strength and the relatively high fraction of micas and clay minerals indicates its thermal stability. The high percentage of quartz and a low percentage of feldspars also an indicator of its chemical stability^94^. The higher amount of ductile minerals such as illite and kaolinite are suggestive of low brittleness index and affirms its integrity to hold CO_2_^95^. The SEM images show pore throat diameters are in the range of 10–40 nm. The capillary entry pressure is inversely proportional to the pore throat diameter; therefore, to flow through these very narrow pore throats, high capillary entry pressure approximately 5–20 MPa will be required^96–98^.

The petrographic and sieve analysis of the Upper Bokabil Sandstone in the Surma Basin suggest that, it is fine to medium grained sand, moderately sorted, fine skewed, leptokurtic and sub arkosic in composition with 18–26% porosity. The abundance of the point contacts along with little line and suture contacts are suggestive of lack of significant diagenesis in the Upper Bokabil Sandstone (Fig. 6). The microscopic image segmentation (Fig. 8B) shows a well-connected pore network.

The petrophysical analysis shows the volume of shale in the Upper Bokabil Sandstones ranges from 17 to 27% which again suggests good reservoir quality. The Thomas Stieber cross plot shows that these shales are distributed predominantly in a laminated manner (Fig. 11). The porosity of the reservoir unit ranges in 23–27% with high permeability 140–860 mD and water saturation ranges from 14 to 21%.

**Figure 19:**
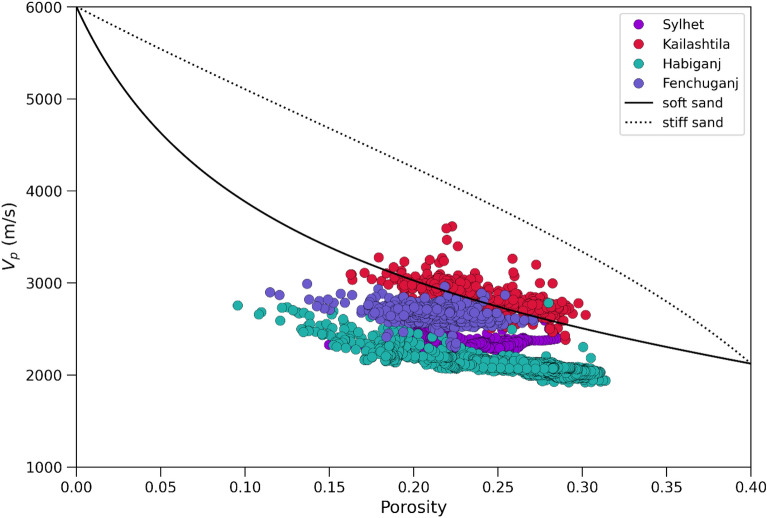
Velocity porosity plot showing in all the fields Upper Bokabil Sandstone is soft in nature and the stiffness is a function of depth.

The differences in the porosity and permeability in the Upper Bokabil Sandstone in the gas fields are mainly due to the consolidation of the sandstones. Velocity-porosity crossplot shows Upper Bokabil Sandstone units of the four structures are soft in nature (Fig. 19). The soft sands are highly porous and exhibit good connectivity among pores^99^. The relative petrophysical analysis among these four structures suggest that, the southern portion of the basin is better on the basis of net reservoir thickness, high effective porosity, low volume of shale, and low water saturation (Table 2). Sandstones in the Kailashtila structure are comparatively stiffer than Sylhet, Habiganj and Fenchuganj structures (Fig. 19) due to the higher burial depth. Hence, they experienced higher burial pressure and compaction, which reduced the primary porosity of the Upper Bokabil Sandstone unit in Kailashtila. The higher burial pressure and compaction lead to diagenetic changes, where feldspars are converted to clay minerals and affect the porosity and permeability^100,101^.

**Figure 20:**
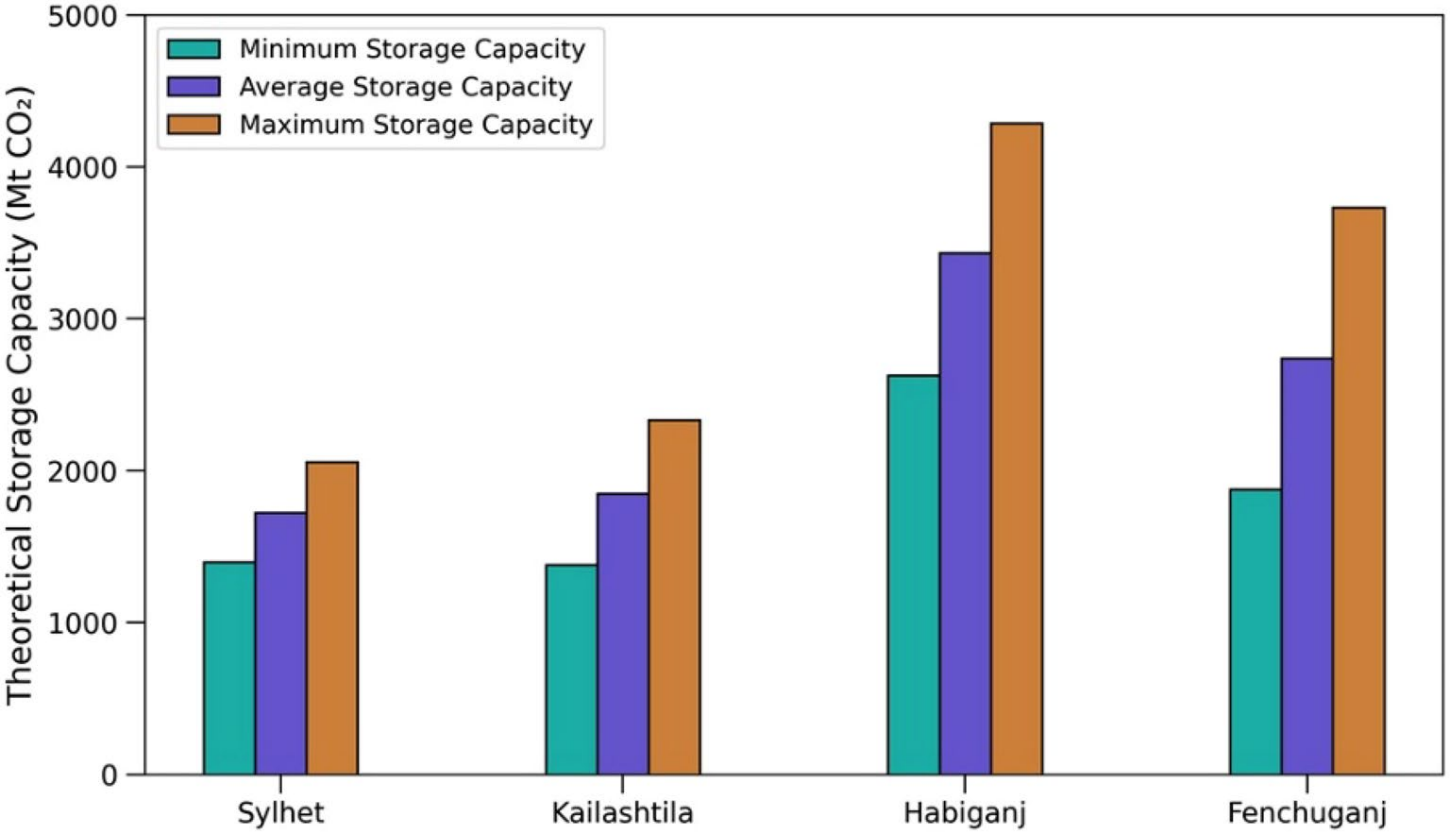
Theoratical storage capacity in all four gas fields. Minimum, average, and maximum storage capacity have been calculated by using CSLF method with varing the petrophysical parameters and areal coverage.

Storage capacity estimated using CLSF method shows that the theoretical storage capacity ranges between 1399–2057 Mt, 1380–2334 Mt, 2624–4285 Mt and 1878–3733 Mt in Sylhet, Kailashtila, Habiganj and Fenchuganj structures, respectively (Fig. 20). The effective storage capacity was estimated by applying the 6% cut off^91^, the effective storage capacity ranges between 84–123 Mt, 83–140 Mt, 157–257 Mt and 113–224 Mt in Sylhet, Kailashtila, Habiganj and Fenchuganj structures, respectively (Fig. 18). These numbers are well above some of the industry scale ongoing CO_2_ projects such as the Sleipner in Norway, In Salah in Algeria, Salt Creek in USA, and Weyburn in Canada. These projects have an effective storage capacity for CO_2_ of 20 Mt, 20 Mt, 27 Mt, and 35 Mt, respectively^102–105^.

Along with good petrophysical properties, the other parameters that dictate how CO_2_ will migrate and trap within the reservoirs are sedimentological heterogeneity^80,106,107^. The presence of small-scale heterogeneity in the form of lithological and facies variability is very important in the CO_2_ storage and trapping as they have significant impact on fluid saturation and relative permeability^106,108–113^. The Upper Bokabil Sandstone unit shows heterogeneity in the lamina scale as well as in the lithofacies scale^53^ (Fig. 12). The presence of mud drapes along the foresets in the crossbedded sandstone and the alternating mudstone and sandstone laminae (Fig. 12) will create capillary equilibrium which might lead to trapping of CO_2_ across the capillary pressure boundary^106,114,115^. Ashraf showed that the mud drapes within sandstone facies act as barriers for CO_2_ plume migration^108^.

To determine the effect of meter scale mud layers (Fig. 12) in the fluid flow through Upper Bokabil Sandstones, the modified equation of Begg and King were utilized to determine the vertical effective permeability^53,83^. Results show that, the effective vertical permeability of the unit can decrease  significantly due to presence of discontinuous mud layers within the Upper Bokabil Sandstones. These mud layers will restrict the vertical flow and force the injected fluids to move laterally beneath the barriers^80,107^. This process will lead to localized trapping of CO_2_ within the reservoir known as local capillary trapping^115,116^. Hovorka et al. performed a series of numerical simulations and suggested in a homogenous reservoir due to the buoyancy of CO_2_, CO_2_ does not disperse and bypass most of reservoir volume which reduces storage capacity^117^. Whereas, stratigraphic heterogeneity can increase CO_2_ sequestration capacity due to dispersive flow network, results distribution of CO_2_ in the whole reservoir^117^. This stratigraphic heterogeneity will enhance the sequestration effectiveness by distributing CO_2_ in the whole reservoir and shortening the height of buoyant CO_2_ plume, which will lead to reduced risk of leakage and seal failure^107,118,119^. The pore volume will be better utilized and the upward migrated CO_2_ will be smaller which will make monitoring of the CO_2_ plume easy and cost effective^81,107^. Therefore, dispersive flow network due to heterogeneity, high horizontal permeability, capability of distribution of CO_2_ in the whole reservoir make the Upper Bokabil Sandstones ideal for carbon storage.

Since the lithological characteristics of any deposits, their porosity, permeability, net reservoir thickness, heterogeneity is controlled by depositional system, the findings from this study can be utilized to better understand the braided fluvial reservoirs in the other basins where they are being developed for CO_2_ storage. Some of the notable ones are Bunter Sandstone Formation in the UK, Buntsandstein Formation in the Netherlands, Utsira Formation in Sleipner field in Norway^120–122^.

The aforementioned characteristics suggest that, the Upper Bokabil Sandstone has good reservoir properties capped by the regional seal rock known as Upper Marine Shale. The high thickness and well connectivity among pores (Fig. 8B) will allow high injectivity of CO_2_. The shallower depth of the Upper Bokabil Sandstones and good petrophysical properties suggest Sylhet and Habiganj structures are more well suited for the initial phase of injection.

## Conclusion

Large scale implementation of Carbon Capture and Storage (CCS) will be required to keep the global warming under control. For this to be successful, identification of good quality storage unit with thick top seals are crucial. This study characterized the Bokabil Formation in the Surma Basin as a potential storage unit. Petrographic analysis showed this is a very good reservoir rock. Having a high thickness, basin-wide lateral extent, higher porosity, and well-connected pores indicate this unit has a significant storage capacity. The presence of laminated shale within the Upper Bokabil Sandstone unit will allow capillary trapping of CO_2_ within the reservoir and regional extent of the top seal Upper Marine Shale offers reliable trapping of CO_2_ in the subsurface for a long time. The estimated cumulative mean effective storage capacity is about 582 Mt CO_2_ in the studied fields, which can be significantly increased if other gas fields in the Surma basin are included in the analysis. The Upper Bokabil Sandstone unit is relatively shallow in the northern and southern parts of the Surma Basin, making them more suitable for storage. The findings in this study would also be applicable to hazardous waste injection, hydrogen storage, etc.

### Supplementary Information

Supplementary Information 1.

## Data Availability

The datasets used and/or analyzed during the current study available from the corresponding author on reasonable request.
